# Extremotolerant fungi from alpine rock lichens and their phylogenetic relationships

**DOI:** 10.1007/s13225-015-0343-8

**Published:** 2015-08-22

**Authors:** Lucia Muggia, Antonia Fleischhacker, Theodora Kopun, Martin Grube

**Affiliations:** Institute of Plant Sciences, University of Graz, Holteigasse 6, 8010 Graz, Austria; Department of Life Sciences, Università degli Studi di Trieste, Via Valerio 12/2, 34128 Trieste, Italy

**Keywords:** Black fungi, Endolichenic, Symbioses, Lichenicolous, Life style, Phylogeny

## Abstract

Fungi other than the lichen mycobiont frequently co-occur within lichen thalli and on the same rock in harsh environments. In these situations dark-pigmented mycelial structures are commonly observed on lichen thalli, where they persist under the same stressful conditions as their hosts. Here we used a comprehensive sampling of lichen-associated fungi from an alpine habitat to assess their phylogenetic relationships with fungi previously known from other niches. The multilocus phylogenetic analyses suggest that most of the 248 isolates belong to the Chaetothyriomycetes and Dothideomycetes, while a minor fraction represents Sordariomycetes and Leotiomycetes. As many lichens also were infected by phenotypically distinct lichenicolous fungi of diverse lineages, it remains difficult to assess whether the culture isolates represent these fungi or are from additional cryptic, extremotolerant fungi within the thalli. Some of these strains represent yet undescribed lineages within Chaethothyriomycetes and Dothideomycetes, whereas other strains belong to genera of fungi, that are known as lichen colonizers, plant and human pathogens, rock-inhabiting fungi, parasites and saprotrophs. The symbiotic structures of the lichen thalli appear to be a shared habitat of phylogenetically diverse stress-tolerant fungi, which potentially benefit from the lichen niche in otherwise hostile habitats.

**Electronic supplementary material:**

The online version of this article (doi:10.1007/s13225-015-0343-8) contains supplementary material, which is available to authorized users.

## Introduction

Bare rock surfaces provide little comfort to life. They are poor sources of nutrients and are constantly exposed to a variety of extremes in abiotic conditions. Variations in surface temperatures and water availability can occur at very short time spans and be the source of diverse stresses (Zakharova et al. 2013; Sterflinger et al. 2012), and with enormous amplitudes. In addition, direct exposure to full sunlight includes a threatening level of energy-rich ultraviolet wavelengths. Not many organisms can cope with such surfaces at this “edge of life”, thus these surfaces are colonized by specialists with particular adaptations (Selbmann et al. 2005, 2013; Onofri et al. 2007; Marzban et al. 2013). In fact, some fungal lineages, which are known as “black fungi” or “microcolonial fungi” are among the most stress-resistant eukaryotic organisms on Earth and can occur at considerable diversity on rocks (Ruibal et al. 2005, 2009). The adaptations of these rock-inhabiting fungi (RIF) include pleomorphic growth, efficient osmolyte management, melanin production, biofilm formation, and survival in cryptobiotic stage (Gostincar et al. 2010, 2011).

Black fungi do not form a monophyletic lineage but are members of Dothideomycetes and Chaetothyriomycetidae (Gueidan et al. 2008; Ruibal et al. 2009) which evolved during periods of dry climate in the late Devonian and middle Triassic, respectively (Gueidan et al. 2011). At approximately the same time scale, the symbiotic association whereby a fungus shelters microscopic algae or cyanobacteria in exchange for fixed carbon and nitrogen helped to ameliorate nutrient deficiencies on rocks. The lichen symbiosis was this key innovation in the evolution of fungi and lichenized mycobionts have since evolved and diversified (Lutzoni et al. 2001; Hawksworth 2015). Particularly, in alpine altitudes where conditions prevent the development of higher plants, lichen thalli express their phenotypic and phylogenetic diversity and shape the landscapes with colorful mosaics on rock surfaces.

In such variably stressed situations, black fungi and lichens can occur side by side on rock, and black fungi also colonize asymptomatic lichens, especially in arid situations (Harutyunyan et al. 2008). Harutyunyan et al. (2008) showed that black fungi may opportunistically infect lichens, but do not cause damage to their host thalli. Some of the fungi resemble hyphomycetous lichenicolous fungi. However, most lichenicolous fungi have a host specific occurrence and are recognized by their phenotypic symptoms and their sexual or asexual spore-producing structures (Hawksworth 1979; Lawrey and Diederich 2003). It is not known whether black fungi, cryptically colonizing lichen thalli, are directly in contact with the photobiont to obtain nutrients. Some studies, however, suggest that black fungi indeed have some affinity to microscopic algae (Brunauer et al. 2007; Gorbushina et al. 2005). Arnold et al. (2009) also used micro-dissection followed by surface sterilization to show that more culturable fungi were associated with the algal layer compared to the medulla and cortex.

In this study, we conducted a comprehensive sampling of saxicolous lichen species (as reported in Fleischhacker et al. 2015), including samples infected by symptoms-developing lichenicolous fungi from different sites of an alpine range, above the tree-line. We prepared culture isolates of the fungi and produced sequence data for phylogenetic analyses. With these we aimed at answering the following questions: i) are there patterns of co-occurrence among cryptic, black extremotolerant fungi, symptomatic lichenicolous fungi and lichen mycobionts?; ii) are lichen-associated fungal communities structured by mycobiont host? ; iii) what is the phylogenetic placement of the isolated strains?

## Material and methods

### Sampling

Lichen thalli were collected on the Koralpe mountain range in the southeastern rim of the Austrian Alps , between the states Styria and Carinthia. The sampling was carried out as in Fleischhacker et al. (2015). Ten collection sites (plots), each further divided into 3 subplots, were selected in alpine habitat, above the timberline, ranging between 1800 and 2100 m a.s.l., and are characterized by big boulders and cliffs of homogeneous size of siliceous-schist/ gneissic rocks separated by wide areas of pastures or dwarf shrub formations. Here winds, in particular from South and West, reach speeds over 120 km/h and the annual temperature averages 0-5 °C (http://www.umwelt.steiermark.at/cms/beitrag/10023583/25206/). In winter, rocks can remain covered by wind-pressed snow and ice for several weeks; alternatively, in summer the south-exposed rock surfaces receive intense solar radiation.

In these sites, boulders’ surfaces are almost entirely colonized by crust-forming (90%), foliose and fruticose (10%) lichens. Crust-forming and foliose lichens were selected for the culture isolation experiment: (i) crustose thalli are composed by contiguous islands of thallus (areoles) which tightly adhere to the substrate with their entire lower surfaces; (ii) foliose thalli adhere only partly to the substrate by a central holdfast (umbilicus) or by root-like appendices (rhizines). About 10% of the lichen thalli in this region are infected by lichenicolous fungi (Fleischhacker et al. 2015). For the isolation of lichenicolous and extremotolerant fungi we selected multiple lichen thalli of different species visibly infected by different species of symptomatic lichenicolous fungi (Tables 1, 2, S3). In doing so, we aimed at including a comprehensive survey of the different lichenicolous fungus-lichen host associations occurring in the area. Within the same subplot, we selected up to four different symptomatically infected thalli. These were either lying close to each other or lying apart up to 50 cm. The lichen thalli were sampled together with their substratum by chiseling the piece of rock. We sampled on both horizontal and vertical positions and at different expositions.

**Table 1:** List of isolates recovered in Eurotiomycetes (Chaetothyriomicetidae). The isolates are identified by their DNA extraction numbers. Number of the original lichen thallus (growth medium of inoculation), name of the lichen, name of the associated lichenicolous fungus, culture collection number and the newly published NCBI accession numbers are reported. Samples in bold are those selected as representatives in the analysis of Fig. 1. The affiliation (clade name) of the other isolates is reported based on the initial analysis including all the isolates. Dash (−) indicate loss of culture due to unsuccessful subsequent growth

Lichen thallus ID (medium name)	Lichen species	Lichenicolous fungus species	Cultured fungus DNA extraction N.	Culture collection N.	nucLSU	nucSSU	mt-SSU	clade ID
**A46 (LBM)**	***Tephromela atra***	***Taeniolella atricerebrina***	**A573**	**LMCC0184**	**KT263034**	**KT263047**	**KT263060**	***clade I***
A46 (SAB)	*Tephromela atra*	*Taeniolella atricerebrina*	A589	LMCC0197	KT263035	KT263048	KT263061	*clade I*
A135 (KGA)	*Lecanora intricata*	*Muellerella* - Li	A515	LMCC0136	KT263033	KT263046	KT263059	*clade I*
**A343 (LBM)**	***Lecanora polytropa***	***Lichenoconium lecanorae***	**A859**	**LMCC0208**	**KT263036**	**KT263049**	**KT263062**	***clade I***
A343 (SAB)	*Lecanora polytropa*	*Lichenoconium lecanorae*	A860	LMCC0233	KT263037	KT263050	KT263063	*clade I*
**A343 (SAB)**	***Lecanora polytropa***	***Lichenoconium lecanorae***	**A861**	**LMCC0234**	**KT263038**	**KT263051**	**KT263064**	***clade I***
**A343 (KGA)**	***Lecanora polytropa***	***Lichenoconium lecanorae***	**A862**	**LMCC0235**	**KT263039**	**KT263052**	**KT263065**	***clade I***
A343 (TM)	*Lecanora polytropa*	*Lichenoconium lecanorae*	A893	LMCC0217	KT263040	KT263053	KT263066	*clade I*
A343 (LBM)	*Lecanora polytropa*	*Lichenoconium lecanorae*	A916	LMCC0265	KT263041	KT263054	KT263067	*clade I*
A343 (TM)	*Lecanora polytropa*	*Lichenoconium lecanorae*	A921	LMCC0266	KT263042	KT263055	KT263068	*clade I*
**A343 (MY)**	***Lecanora polytropa***	***Lichenoconium lecanorae***	**A922**	**LMCC0267**	**KT263043**	**KT263056**	**KT263069**	***clade I***
A343 (KGA)	*Lecanora polytropa*	*Lichenoconium lecanorae*	A936	LMCC0276	KT263044	KT263057	KT263070	*clade I*
**A666 (SAB)**	***Rhizocarpon geographicum***	***Endococcus macrosporus***	**A1022**	**LMCC0346**	**KT263045**	**KT263058**	**KT263071**	***clade I***
**A97 (KGA)**	***Rhizocarpon geographicum***	***Muellerella*** **- Rh**	**A944**	**LMCC0283**	**KT263072**	**KT263094**	**KT263110**	***clade II***
A97 (KGA)	*Rhizocarpon geographicum*	*Muellerella* - Rh	A994	LMCC0332	KT263074	–	KT263112	*clade II*
**A263 (SAB)**	***Rhizocarpon geographicum***	***Muellerella*** **- Rh**	**A993**	**LMCC0331**	**KT263073**	**KT263095**	**KT263111**	***clade II***
A385 (TMY)	*Rhizocarpon geographicum*	*Muellerella* - Rh	A1003	LMCC0364	KT263075	–	KT263113	*clade II*
**A385 (MY)**	***Rhizocarpon geographicum***	***Muellerella*** **- Rh**	**A1015**	**LMCC0340**	**KT263076**	**KT263096**	**KT263114**	***clade II***
**A46 (SAB)**	***Tephromela atra***	***T. atricerebrina*** **(+** ***Miutoexcipula tephromelae*** **)**	**A528**	**LMCC0148**	**KT263088**	**KT263104**	**KT263123**	***clade III***
	***Tephromela atra***	***Lichenodiplis lecanorae***	**L1858**	**–**	**KT263086**	**KT263100**	**KT263118**	***clade III***
	***Tephromela atra***	***Lichenodiplis lecanorae***	**L1860**	**LMCC0513**	**KT263087**	**KT263101**	**KT263119**	***clade III***
	***Tephromela atra***	***Muellerella atricola***	**L1992**	**LMCC0066**	**KT263083**	**–**	**KT263120**	***clade III***
	***Tephromela atra***	***Muellerella atricola***	**L1993**	**LMCC0487**	**KT263084**	**KT263102**	**KT263121**	***clade III***
	***Tephromela atra***	***Muellerella atricola***	**L1994**	**LMCC0515**	**KT263085**	**KT263103**	**KT263122**	***clade III***
**A64 (TM)**	***Schaereria fuscocinerea***	***Endococcus perpusillus***	**A511**	**LMCC0132**	**KT263126**	**KT263171**	**KT263215**	**–**
A64 (LBM)	*Schaerera fuscocinerea*	*Endococcus perpusillus*	A570	LMCC0181	KT263158	KT263202	KT263246	–
A65 (TM)	*Lecanora polytropa*	*Muellerella* - Lp	A891	LMCC0253	KT270616	KT270704	KT270786	–
**A72 (SAB)**	***Lecanora polytropa***	***Carbonea supersparsa***	**A514**	**LMCC0135**	**KT263128**	**KT263173**	**KT263217**	**–**
**A100 (MY)**	***Umbilicaria cylindrica***	***Stigmidium gyrophorarum***	**A584**	**LMCC0193**	**KT263164**	**KT263208**	**KT263252**	**–**
A198 (KGA)	*Lecanora polytropa*	*Muellerella* - Lp	A961	LMCC0311	KT270661	–	KT270830	–
A198 (KGA)	*Lecanora polytropa*	*Muellerella* - Lp	A1010	LMCC0367	KT263332	KT263365	KT263397	–
**A267 (KGA)**	***Aspicilia***	***Endococcus verrucosus***	**A885**	**LMCC0248**	**KT270614**	**KT270702**	**KT270784**	**–**
A267 (TM)	*Aspicilia*	*Endococcus verrucosus*	A903	LMCC0223	KT270624	KT270712	KT270794	–
**A267 (TM)**	***Aspicilia***	***Endococcus verrucosus***	**A911**	**LMCC0230**	**KT270629**	**KT270717**	**KT270799**	**–**
A267 (TM)	*Aspicilia*	*Endococcus verrucosus*	A949	LMCC0286	KT270653	KT2707238	KT270822	–
**A319 (TM)**	***Lecidea sp.***	***Muellerella pygmaea***	**A873**	**LMCC0242**	**KT270602**	**KT270690**	**KT270772**	**–**
A319 (SAB)	*Lecidea sp.*	*Muellerella pygmaea*	A875	LMCC0264	KT270604	KT270692	KT270774	–
**A329 (TM)**	***Aspicilia***	***Endococcus verrucosus***	**A924**	LMCC0261	**KT270635**	**KT270724**	**KT270804**	–
**A329 (LBM)**	***Aspicilia***	***Endococcus verrucosus***	**A939**	LMCC0278	**KT270644**	**–**	**KT270813**	–
**A347 (TM)**	***Lecidea lapicida***	***Cecidonia umbonella***	**A865**	**LMCC0238**	**KT270594**	**KT270682**	**KT270764**	**–**
A347 (KGA)	*Lecidea lapicida*	*Cecidonia umbonella*	A866	LMCC0239	KT270595	KT270683	KT270765	–
**A390 (LBM)**	***Rhizocarpon geographicum***	***Endococcus macrosporus***	**A956**	**LMCC0292**	**KT270658**	**KT270742**	**KT270827**	**–**
**A440 (MY)**	***Tephromela atra***	***Muellerella atricola***	**A1053**	**LMCC0385**	**KT263356**	**KT263388**	**KT263420**	**–**
**A23 (TM)**	***Lecanora intricata***	***Muellerella*** **- Li**	**A989**	**LMCC0330**	**KT270678**	**KT270760**	**KT270847**	***clade IV***
A65 (TM)	*Lecanora polytropa*	*Muellerella* - Lp	A516	LMCC0137	KT263130	KT263174	KT263218	*clade IV*
**A65 (TM)**	***Lecanora polytropa***	***Muellerella*** **- Lp**	**A522**	**LMCC0142**	**KT263134**	**KT263178**	**KT263222**	***clade IV***
A65 (LBM)	*Lecanora polytropa*	*Muellerella* - Lp	A531	LMCC0150	KT263140	KT263184	KT263228	*clade IV*
**A65 (SAB)**	***Lecanora polytropa***	***Muellerella*** **- Lp**	**A532**	**LMCC0151**	**KT263141**	**KT263185**	**KT263229**	***clade IV***
**A65 (MY)**	***Lecanora polytropa***	***Muellerella*** **- Lp**	**A539**	**LMCC0157**	**KT263146**	**KT263190**	**KT263234**	***clade IV***
A65 (TM)	*Lecanora polytropa*	*Muellerella* - Lp	A541	LMCC0158	KT263147	KT263191	KT263235	*clade IV*
A65 (SAB)	*Lecanora polytropa*	*Muellerella* - Lp	A547	–	KT263151	KT263195	KT263239	*clade IV*
A65 (LBM)	*Lecanora polytropa*	*Muellerella* - Lp	A548	LMCC0163	KT263152	KT263196	KT263240	*clade IV*
A84 (MY)	*Lecanora polytropa*	*Lichenoconium lecanorae*	A517	LMCC0138	KT263131	KT263175	KT263219	*clade IV*
A84 (TM)	*Lecanora polytropa*	*Lichenoconium lecanorae*	A520	LMCC0140	KT263132	KT263176	KT263220	*clade IV*
**A84 (TM)**	***Lecanora polytropa***	***Lichenoconium lecanorae***	**A533**	**LMCC0191**	**KT263142**	**KT263186**	**KT263230**	***clade IV***
A84 (LBM)	*Lecanora polytropa*	*Lichenoconium lecanorae*	A909	LMCC0228	KT270628	KT270716	KT270798	*clade IV*
**A135 (TM)**	***Lecanora polytropa***	***Muellerella*** **- Lp**	**A950**	**LMCC0287**	**KT270654**	**KT270739**	**KT270823**	***clade IV***
A237 (LBM)	*Rhizocarpon geographicum*	*Muellerella* - Rh	A895	LMCC0219	KT270619	KT270707	KT270789	*clade IV*
**A237 (KGA)**	***Rhizocarpon geographicum***	***Muellerella*** **- Rh**	**A929**	**LMCC0262**	**KT270640**	**KT270728**	**KT270809**	***clade IV***
A194 (LBM)	*Rhizocarpon geographicum*	*Endococcus macrosporus*	A889	LMCC0251	KT270615	KT270703	KT270785	*clade IV*
**A194 (SAB)**	***Rhizocarpon geographicum***	***Endococcus macrosporus***	**A918**	**LMCC0361**	**KT270632**	**KT270721**	**KT270801**	***clade IV***
**A254 (SAB)**	***Lecanora polytropa***	***Muellerella*** **- Lp**	**A1045**	**LMCC0377**	**KT263349**	**KT263381**	**KT263413**	***clade IV***
**A675 (KGA)**	***Tephromela atra***	***Taeniolella atricerebrina***	**A980**	LMCC0317	**KT270672**	**KT270754**	**KT270841**	***clade IV***
**A832 (KGA)**	***Lecanora bicincta***	***Arthonia varians***	**A969**	**LMCC0300**	**KT270665**	**KT270748**	**KT270834**	***clade IV***
**A94 (LBM)**	***Lecanora intricata***	***Muellerella*** **- Li**	**A587**	**LMCC0195**	**KT263165**	**KT263209**	**KT263253**	***clade V***
A122 (MY)	*Aspicilia caesiocinerea*	*Endococcus rugulosus*	A521	LMCC0141	KT263133	KT263177	KT263221	*clade V*
**A122 (LBM)**	***Aspicilia caesiocinerea***	***Endococcus rugulosus***	**A574**	**LMCC0185**	**KT263160**	**KT263204**	**KT263248**	***clade V***
**A267 (MY)**	***Aspicilia***	***Endococcus verrucosus***	**A952**	**LMCC0289**	**KT270655**	**–**	**KT270824**	***clade V***
A267 (SAB)	*Aspicilia*	*Endococcus verrucosus*	A1000	LMCC0333	KT263328	KT263361	KT263393	*clade V*
A307 (LBM)	*Lecanora intricata*	*Muellerella* - Li	A879	LMCC0246	KT270608	KT270696	KT270778	*clade V*
**A307 (LBM)**	***Lecanora intricata***	***Muellerella*** **- Li**	**A884**	**LMCC0247**	**KT270613**	**KT270701**	**KT270783**	***clade V***
A309 (LBM)	*Rhizocarpon geographicum*	*Endococcus macrosporus*	A912	LMCC0256	KT270630	KT270718	KT270800	*clade V*
**A309 (TM)**	***Rhizocarpon geographicum***	***Endococcus macrosporus***	**A914**	**LMCC0257**	**KT270631**	**KT270719**	**–**	***clade V***
A309 (SAB)	*Rhizocarpon geographicum*	*Endococcus macrosporus*	A920	LMCC0260	KT270634	KT270723	KT270803	*clade V*
**A329 (TM)**	***Aspicilia***	***Endococcus verrucosus***	**A926**	**LMCC0304**	**KT270637**	**KT270726**	**KT270806**	***clade V***
A329 (TM)	*Aspicilia*	*Endococcus verrucosus*	A927	LMCC0270	KT270638	–	KT270807	*clade V*
A329 (LBM)	*Aspicilia*	*Endococcus verrucosus*	A928	LMCC0271	KT270639	KT270727	KT270808	*clade V*
**A352 (KGA)**	***Lecanora polytropa***	***Cercidospora epipolytropa***	**A945**	**LMCC0284**	**KT270649**	**KT270735**	**KT270818**	***clade V***
A352 (TM)	*Lecanora polytropa*	*Cercidospora epipolytropa*	A946	LMCC0307	KT270650	KT270736	KT270819	*clade V*
**A398 (SAB)**	***Lecidea lapicida***	***Cecidonia umbonella***	**A955**	**LMCC0291**	**KT270657**	**KT270741**	**KT270826**	***clade V***
**A666 (SAB)**	***Rhizocarpon geographicum***	***Endococcus macrosporus***	**A1025**	**LMCC0348**	**KT263337**	**KT263370**	**–**	***clade V***
**A703 (SAB)**	***Lecanora polytropa***	***Muellerella*** **- Lp**	**A983**	**LMCC0302**	**KT270674**	**KT270756**	**KT270843**	***clade V***
A703 (LBM)	*Lecanora polytropa*	*Muellerella* - Lp	A987	LMCC0321	KT270676	KT270758	KT270845	*clade V*
A703 (LBM)	*Lecanora polytropa*	*Muellerella* - Lp	A974	LMCC0314	KT270668	KT270751	KT270837	*clade V*
**A12 (MY)**	***Lecidea sp.***	***Muellerella pygmaea***	**A526**	**LMCC0146**	**KT263136**	**KT263180**	**KT263224**	***clade VI***
A12 (SAB)	*Lecidea sp.*	*Muellerella pygmaea*	A527	LMCC0147	KT263137	KT263181	KT263225	*clade VI*
A12 (TM)	*Lecidea sp.*	*Muellerella pygmaea*	A530	–	KT263139	KT263183	KT263227	*clade VI*
A12 (TM)	*Lecidea sp.*	*Muellerella pygmaea*	A535	LMCC0153	KT263143	KT263187	KT263231	*clade VI*
A12 (SAB)	*Lecidea sp.*	*Muellerella pygmaea*	A536	LMCC0154	KT263144	KT263188	KT263232	*clade VI*
**A12 (LBM)**	***Lecidea sp.***	***Muellerella pygmaea***	**A544**	**LMCC0161**	**KT263149**	**KT263193**	**KT263237**	***clade VI***
A12 (LBM)	*Lecidea sp.*	*Muellerella pygmaea*	A546	–	KT263150	KT263194	KT263238	*clade VI*
A12 (LBM)	*Lecidea sp.*	*Muellerella pygmaea*	A892	LMCC0216	KT270617	KT270705	KT270787	*clade VI*
A37 (TM)	*Tephromela atra*	*Taeniolella atricerebrina*	A898	–	KT270621	KT270709	KT270791	*clade VI*
A46 (LBM)	*Tephromela atra*	*Taeniolella atricerebrina*	A525	LMCC0393	KT263135	**KT263179**	**KT263223**	*clade VI*
**A46 (SAB)**	***Tephromela atra***	***Taeniolella atricerebrina***	**A572**	**LMCC0183**	**KT263159**	**KT263203**	**KT263225**	***clade VI***
A94 (SAB)	*Lecanora intricata*	*Muellerella* -Li	A575	LMCC0186	KT263161	**KT263205**	**KT263249**	*clade VI*
**A94 (LBM)**	***Lecanora intricata***	***Muellerella*** **- Li**	**A576**	**LMCC0187**	**KT263162**	**KT263206**	**KT263250**	***clade VI***
A94 (MY)	*Lecanora intricata*	*Muellerella* -Li	A581	LMCC0192	KT263163	KT263207	KT263251	*clade VI*
A100 (TM)	*Umbilicaria cylindrica*	*Stigmidium gyrophorarum*	A564	LMCC0175	KT263155	KT263199	KT263243	*clade VI*
**A100 (KGA)**	***Umbilicaria cylindrica***	***Stigmidium gyrophorarum***	**A566**	**LMCC0177**	**KT263156**	**KT263200**	**KT263244**	***clade VI***
A106 (SAB)	*Tephromela atra*	*Taeniolella atricerebrina*	A562	LMCC0173	KT263154	KT263198	KT263242	*clade VI*
A149 (TM)	*Protoparmela badia*	*Phacographa protoparmeliae*	A555	LMCC0166	KT263153	KT263197	KT263241	*clade VI*
**A173 (SAB)**	***Lecanora polytropa***	***Lichenoconium lecanorae***	**A513**	**LMCC0134**	**KT263127**	**KT263172**	**KT263216**	***clade VII***
A173 (SAB)	*Lecanora polytropa*	*Lichenoconium lecanorae*	A529	LMCC0149	KT263138	KT263182	KT263226	*clade VII*
**A173 (TM)**	***Lecanora polytropa***	***Lichenoconium lecanorae***	**A538**	**LMCC0156**	**KT263145**	**KT263189**	**KT263233**	***clade VII***
A173 (KGA)	*Lecanora polytropa*	*Lichenoconium lecanorae*	A943	LMCC0282	KT270648	**KT270734**	KT270817	*clade VII*
**A184 (MY)**	***Tephromela atra***	***Taeniolella atricerebrina***	**A543**	**LMCC0160**	**KT263148**	**KT263192**	**KT263236**	***clade VI***
A184 (SAB)	*Tephromela atra*	*Taeniolella atricerebrina*	A596	LMCC0203	KT263167	KT263211	KT263255	*clade VI*
A184 (LBM)	*Tephromela atra*	*Taeniolella atricerebrina*	A597	LMCC0204	KT263168	KT263212	KT263256	*clade VI*
**A184 (KGA)**	***Tephromela atra***	***Taeniolella atricerebrina***	**A599**	**LMCC0206**	**KT263170**	**KT263214**	**KT263258**	***clade VI***
**A198 (TM)**	***Rhizocarpon geographicum***	***Endococcus macrosporus***	**A933**	**LMCC0274**	**KT270641**	**–**	**KT270810**	***clade VII***
A215 (KGA)	*Lecanora polytropa*	*Lichenoconium lecanorae*	A598	LMCC0205	KT263169	KT263213	KT263257	*clade VI*
**A215 (TM)**	***Lecanora polytropa***	***Lichenoconium lecanorae***	**A594**	**LMCC0201**	**KT263166**	**KT263210**	**KT263254**	***clade VI***
**A241 (KGA)**	***Tephromela atra***	***Taeniolella atricerebrina***	**A894**	**LMCC0218**	**KT270618**	**KT270706**	**KT270788**	***clade VI***
A254 (LBM)	*Lecanora polytropa*	*Muellerella* - Lp	A906	LMCC0226	KT270627	KT270715	KT270797	*clade VI*
**A254 (KGA)**	***Lecanora polytropa***	***Muellerella - Lp***	**A953**	**LMCC0309**	**KT270656**	**KT270740**	**KT270825**	***clade VI***
A254 (SAB)	*Lecanora polytropa*	*Muellerella* - Lp	A962	LMCC0296	KT270662	KT270745	KT270831	*clade VI*
**A263 (LBM)**	***Rhizocarpon geographicum***	***Muellerella*** **- Rh**	**A878**	**LMCC0245**	**KT270607**	**KT270695**	**KT270777**	***clade VI***
A280 (KGA)	*Tephromela atra*	*Skyttea tephromelarum*	A880	LMCC0212	KT270609	KT270697	KT270779	*clade VI*
**A280 (MY)**	***Tephromela atra***	***Skyttea tephromelarum***	**A881**	**LMCC0213**	**KT270610**	**KT270698**	**KT270780**	***clade VI***
A280 (LBM)	*Tephromela atra*	*Skyttea tephromelarum*	A882	LMCC0214	KT270611	KT270699	KT270781	*clade VI*
A280 (LBM)	*Tephromela atra*	*Skyttea tephromelarum*	A883	LMCC0215	KT270612	KT270697	KT270782	*clade VI*
A280 (SAB)	*Tephromela atra*	*Skyttea tephromelarum*	A896	–	KT270620	KT270708	KT270790	*clade VI*
**A280 (TM)**	***Tephromela atra***	***Skyttea tephromelarum***	**A900**	**LMCC0220**	**KT270622**	**KT270710**	**KT270792**	***clade VI***
A280 (SAB)	*Tephromela atra*	*Skyttea tephromelarum*	A942	LMCC0281	KT270647	**KT270733**	KT270816	*clade VI*
**A296 (MY)**	***Rhizocarpon geographicum***	***Endococcus macrosporus***	**A934**	LMCC0275	**KT270642**	**KT270729**	**KT270811**	***clade VI***
A296 (SAB)	*Rhizocarpon geographicum*	*Endococcus macrosporus*	A948	LMCC0308	KT270652	**KT270737**	KT270821	*clade VI*
**A319 (SAB)**	***Lecidea sp.***	***Muellerella pygmaea***	**A869**	**LMCC0263**	**KT270598**	**KT270686**	**KT270768**	***clade VI***
A319 (MY)	*Lecidea sp.*	*Muellerella pygmaea*	A874	LMCC0243	KT270603	KT270691	KT270773	*clade VI*
A319 (TM)	*Lecidea sp.*	*Muellerella pygmaea*	A938	LMCC0277	KT270643	**KT270730**	KT270812	*clade VI*
**A319 (TM)**	***Lecidea sp.***	***Muellerella pygmaea***	**A940**	LMCC0279	**KT270645**	**KT270731**	**KT270814**	***clade VI***
A319 (SAB)	*Lecidea sp.*	*Muellerella pygmaea*	A941	LMCC0280	KT270646	**KT270732**	**KT2708145**	*clade VI*
A319 (LBM)	*Lecidea sp.*	*Muellerella pygmaea*	A998	LMCC0325	KT270670	**KT270761**	**KT270848**	*clade VI*
A329 (MY)	*Aspicilia*	*Endococcus verrucosus*	A925	LMCC0269	KT270636	**KT270725**	**KT270805**	*clade VI*
A329 (LBM)	*Aspicilia*	*Endococcus verrucosus*	A947	LMCC0285	KT270651	–	**KT270820**	*clade VI*
**A329 (KGA)**	***Aspicilia***	***Endococcus verrucosus***	**A1013**	**LMCC0338**	**KT263333**	**KT263366**	**KT263398**	***clade VI***
A347 (TM)	*Lecidea lapicida*	*Cecidonia umbonella*	A864	LMCC0237	KT270593	KT270681	KT270763	*clade VI*
**A347 (KGA)**	***Lecidea lapicida***	***Cecidonia umbonella***	**A867**	**LMCC0240**	**KT270596**	**KT270684**	**KT270766**	***clade VI***
A347 (KGA)	*Lecidea lapicida*	*Cecidonia umbonella*	A868	LMCC0209	KT270597	KT270685	KT270767	*clade VI*
A347 (LBM)	*Lecidea lapicida*	*Cecidonia umbonella*	A870	LMCC0210	KT270599	KT270687	KT270769	*clade VI*
A347 (SAB)	*Lecidea lapicida*	*Cecidonia umbonella*	A871	LMCC0211	KT270600	KT270688	KT270770	*clade VI*
**A347 (LBM)**	***Lecidea lapicida***	***Cecidonia umbonella***	**A872**	**LMCC0241**	**KT270601**	**KT270689**	**KT270771**	***clade VI***
A347 (TM)	*Lecidea lapicida*	*Cecidonia umbonella*	A876	–	KT270605	KT270693	KT270775	*clade VI*
A347 (KGA)	*Lecidea lapicida*	*Cecidonia umbonella*	A877	LMCC0244	KT270606	KT270694	KT270776	*clade VI*
A357 (TM)	*Lecidea sp.*	*Muellerella pygmaea*	A904	LMCC0224	KT270625	KT270713	KT270795	*clade VI*
A357 (TM)	*Lecidea sp.*	*Muellerella pygmaea*	A905	LMCC0225	KT270626	KT270714	KT270796	*clade VI*
**A357 (SAB)**	***Lecidea sp.***	***Muellerella pygmaea***	**A958**	**LMCC0294**	**KT270660**	**KT270744**	**KT270829**	***clade VI***
A357 (LBM)	*Lecidea sp.*	*Muellerella pygmaea*	A1002	LMCC0334	KT263329	KT263362	KT263394	*clade VI*
A357 (KGA)	*Lecidea sp.*	*Muellerella pygmaea*	A1004	LMCC0335	KT263330	KT263363	KT263395	*clade VI*
A357 (LBM)	*Lecidea sp.*	*Muellerella pygmaea*	A1018	LMCC0342	KT263334	KT263367	KT263399	*clade VI*
**A357 (TM)**	***Lecidea sp.***	***Muellerella pygmaea***	**A1019**	**LMCC0343**	**KT263335**	**KT263368**	**KT263400**	***clade VI***
A373 (KGA)	*Lecidea sp.*	*Muellerella pygmaea*	A957	LMCC0293	KT270659	KT270743	KT270828	*clade VI*
A373 (TM)	*Lecidea sp.*	*Muellerella pygmaea*	A1030	LMCC0352	KT263340	KT263373	KT263404	*clade VI*
A408 (LBM)	*Rhizocarpon geographicum*	*Endococcus macrosporus*	A901	LMCC0221	KT270623	KT270711	KT270793	*clade VI*
**A408 (TM)**	***Rhizocarpon geographicum***	***Endococcus macrosporus***	**A919**	**LMCC0259**	**KT270633**	**KT270722**	**KT270802**	***clade VII***
**A426 (KGA)**	***Lecanora polytropa***	***Muellerella*** **- Lp**	**A1049**	**LMCC0381**	**KT263352**	**KT263384**	**KT263416**	***clade VII***
A440 (SAB)	*Tephromela atra*	*Muellerella atricola*	A1050	LMCC0382	KT263353	KT263385	KT263417	*clade VII*
**A440 (LBM)**	***Tephromela atra***	***Muellerella atricola***	**A1051**	**LMCC0383**	**KT263354**	**KT263386**	**KT263418**	***clade VII***
A440 (LBM)	*Tephromela atra*	*Muellerella atricola*	A1052	LMCC0384	KT263355	KT263387	KT263419	*clade VII*
**A440 (LBM)**	***Tephromela atra***	***Muellerella atricola***	**A1054**	**LMCC0386**	**KT263357**	**KT263389**	**–**	***clade VII***
**A464 (LBM)**	***Tephromela atra***	***Skyttea tephromelarum***	**A1058**	**LMCC0390**	**KT263358**	**KT263390**	**KT263421**	***clade VI***
A464 (MY)	*Tephromela atra*	*Skyttea tephromelarum*	A1059	LMCC0391	KT263359	KT263391	KT263422	*clade VI*
**A469 (SAB)**	***Rhizocarpon geographicum***	***Opegrapha geographicola***	**A1060**	**–**	**KT263360**	**KT263392**	**KT263423**	***clade VI***
**A475 (LBM)**	***Tephromela atra***	***Taeniolella atricerebrina***	**A1008**	**LMCC0337**	**KT263331**	**KT263364**	**KT263396**	***clade VI***
**A613 (KGA)**	***Schaereria fuscocinerea***	***Muellerella*** **- Sf**	**A986**	**LMCC0320**	**KT270675**	**KT270757**	**KT270844**	***clade VI***
**A643 (KGA)**	***Lecidea sp.***	***Muellerella pygmaea***	**A1029**	**LMCC0351**	**KT263339**	**KT263372**	**KT263403**	***clade VI***
A651 (TM)	*Lecanora polytropa*	*Carbonea supersparsa*	A1020	LMCC0344	KT263336	KT263369	KT263401	*clade VI*
**A651 (KGA)**	***Lecanora polytropa***	***Carbonea supersparsa***	**A1046**	**LMCC0378**	**KT263350**	**KT263382**	**KT263414**	***clade VI***
**A653 (MY)**	***Tephromela atra***	***Taeniolella atricerebrina***	**A975**	**LMCC0315**	**KT270669**	**KT270752**	**KT270838**	***clade VI***
**A663 (LBM)**	***Tephromela atra***	***Muellerella atricola***	**A981**	**LMCC0318**	**KT270673**	**KT270755**	**KT270842**	***clade VI***
A663 (TM)	*Tephromela atra*	*Muellerella atricola*	A1042	LMCC0375	KT263348	–	KT263412	*clade VI*
**A670 (KGA)**	***Lecanora polytropa***	***Muellerella*** **- Lp**	**A999**	**LMCC0326**	**KT270680**	**KT270762**	**KT270849**	***clade VI***
A670 (TM)	*Lecanora polytropa*	*Muellerella* - Lp	A1035	–	KT263344	KT263377	KT263408	*clade VI*
**A683 (SAB)**	***Lecanora polytropa***	***Muellerella*** **- Lp**	**A978**	LMCC0301	**KT270670**	**KT270753**	**KT270839**	***clade VI***
A683 (LBM)	*Lecanora polytropa*	*Muellerella* - Lp	A988	LMCC0303	KT270677	KT270759	KT270846	*clade VI*
A683 (TM)	*Lecanora polytropa*	*Muellerella* - Lp	A1036	LMCC0357	KT263345	KT263378	KT263409	*clade VI*
**A683 (TM)**	***Lecanora polytropa***	***Muellerella*** **- Lp**	**A1047**	**LMCC0379**	**KT263351**	**KT263383**	**KT263415**	***clade VI***
A689 (SAB)	*Tephromela atra*	*Taeniolella atricerebrina*	A979	LMCC0316	KT270671	–	KT270840	*clade VI*
**A698 (KGA)**	***Rhizocarpon geographicum***	***Endococcus macrosporus***	**A971**	LMCC0313	**KT270666**	**KT270749**	**KT270835**	***clade VI***
A698 (MY)	*Rhizocarpon geographicum*	*Endococcus macrosporus*	A1027	LMCC0349	KT263338	KT263371	KT263402	*clade VI*
**A703 (TM)**	***Lecanora polytropa***	***Muellerella*** **- Lp**	**A1031**	**LMCC0353**	**KT263341**	**KT263374**	**KT263405**	***clade VI***
A703 (TM)	*Lecanora polytropa*	*Muellerella* - Lp	A1032	LMCC0354	KT263342	KT263375	KT263406	*clade VI*
A703 (MY)	*Lecanora polytropa*	*Muellerella* - Lp	A1034	LMCC0356	KT263343	KT263376	KT263407	*clade VI*
**A703 (MY)**	***Lecanora polytropa***	***Muellerella*** **- Lp**	**A1040**	**LMCC0373**	**KT263346**	**KT263379**	**KT263410**	***clade VI***
**A709 (MY)**	***Rhizocarpon geographicum***	***Muellerella*** **- Rh**	**A1041**	**LMCC0374**	**KT263347**	**KT263380**	**KT263411**	***clade VI***
**A832 (MY)**	***Lecanora bicincta***	***Arthonia varians***	**A967**	LMCC0312	**KT270663**	**KT270746**	**KT270832**	***clade VI***
**A840 (TM)**	***Lecidea sp.***	***Muellerella pygmaea***	**A968**	LMCC0299	**KT270664**	**KT270747**	**KT270833**	***clade VI***
A840 (TM)	*Lecidea sp.*	*Muellerella pygmaea*	A973	LMCC0396	KT270667	KT270750	KT270836	*clade VI*
**A341 (TM)**	***Pertusaria corallina***	***Sclaerococcum sphaerale***	**A1016**	**LMCC0341**	**KT263077**	**KT263097**	**KT263115**	***Sclerococcum***
**A100 (SAB)**	***Umbilicaria cylindrica***	***Stigmidium gyrophorarum***	**A561**	**LMCC0172**	**KT263079**	**KT263092**	**KT263108**	**basal to Chaetothyriaceae**
A100 (LBM)	*Umbilicaria cylindrica*	*Stigmidium gyrophorarum*	A563	LMCC0174	KT263080	KT263093	KT263109	basal to Chaetothyriaceae
**A94 (KGA)**	***Lecanora intricata***	***Muellerella*** **- In**	**A512**	**LMCC0133**	**KT263078**	**KT263091**	**KT263107**	**Herpothrychielaceae**
**A670 (LBM)**	***Lecanora polytropa***	***Muellerella*** **- Lp**	**A1044**	**LMCC0376**	**KT263082**	**KT263099**	**KT263117**	**Herpothrychielaceae**
**A678 (SAB)**	***Lecanora bicincta***	***Sphaerellothecium atrinae***	**A1033**	**LMCC0355**	**KT263081**	**KT263098**	**KT263116**	**Herpothrychielaceae**
**A97 (KGA)**	***Rhizocarpon geographicum***	***Muellerella*** **- Rh**	**A579**	**–**	**KT263089**	**KT263105**	**KT263124**	**Epibryaceae**
**A651 (LBM)**	***Lecanora polytropa***	***Carbonea supersparsa***	**A1026**	**LMCC0370**	**KT263090**	**KT263106**	**KT263125**	**Epibryaceae**
**A56 (TM)**	***Lecanora intricata***	***Muellerella*** **- Li**	**A568**	**LMCC0179**	**KT263157**	**KT263201**	**KT263245**	**incerta saedis**

**Table 2:** List of isolates recovered in Dothideomycetes as in the phylogenetic analysis of Fig. 3. The isolates are identified by their DNA extraction numbers. Number of the original lichen thallus (growth medium of inoculation), name of the lichen, name of the associated lichenicolous fungus, culture collection number and the newly published NCBI accession numbers are reported. The affiliation (clade name) of the isolates is reported. Dash (−) indicate loss of culture due to unsuccessful subsequent growth

Lichen thallus ID (medium name)	Lichen species	Lichenicolous fungus species	Cultured fungus DNA extraction N.	Culture collection N.	LSU	nuSSU	mtSSU	Phlogenetic clade
A56 (LBM)	*Lecanora intricata*	*Muellerella* - Li	A545	LMCC0162	**KT263458**	**KT263493**	**KT263528**	Capnodiales
A56 (TM)	*Lecanora intricata*	*Muellerella* - Li	A571	LMCC0182	**KT263457**	**KT263492**	**KT263527**	Capnodiales
A56 (SAB)	*Lecanora intricata*	*Muellerella* - Li	A577	LMCC0188	**KT263459**	**KT263494**	**KT263529**	Capnodiales
A56 (SAB)	*Lecanora intricata*	*Muellerella* - Li	A923	LMCC0268	**KT263456**	**KT263491**	**KT263526**	Capnodiales
A56 (MY)	*Lecanora intricata*	*Muellerella* - Li	A959	LMCC0310	**KT263460**	**KT263487**	**KT263522**	Capnodiales
A102 (DG)	*Acarospora fuscata*	*Polycoccum microstictum*	A557	LMCC0168	**KT263447**	**KT263481**	**KT263516**	Capnodiales
A102 (DG)	*Acarospora fuscata*	*Polycoccum microstictum*	A951	LMCC0288	**KT263448**	**KT263482**	**KT263517**	Capnodiales
A135 (LBM)	*Lecanora polytropa*	*Muellerella* - Lp	A886	–	**KT263453**	**KT263488**	**KT263523**	Capnodiales
A135 (KGA)	*Lecanora polytropa*	*Muellerella* - Lp	A887	LMCC0249	**KT263454**	**KT263489**	**KT263524**	Capnodiales
A135 (SAB)	*Lecanora polytropa*	*Muellerella* - Lp	A888	LMCC0250	**KT263455**	**KT263490**	**KT263525**	Capnodiales
A215 (SAB)	*Lecanora polytropa*	*Lichenoconium lecanorae*	A913	–	**KT263450**	**KT263484**	**KT263519**	Capnodiales
A224 (DG)	*Lecanora polytropa*	*Carbonea supersparsa*	A863	LMCC0236	**KT263451**	**KT263485**	**KT263520**	Capnodiales
A229 (DG)	*Lecanora polytropa*	*Carbonea supersparsa*	A997	LMCC0324	**KT263449**	**KT263483**	**KT263518**	Capnodiales
A291 (DG)	*Lecanora rupicola*	*Arthonia varians*	A995	LMCC0362	**KT263445**	**KT263479**	**–**	Capnodiales
A393 (KGA)	*Lecanora polytropa*	*Cercidospora epipolytropa*	A960	LMCC0295	**KT263452**	**KT263486**	**KT263521**	Capnodiales
A709 (MY)	*Rhizocarpon geographicum*	*Muellerella* - Rh	A1043	LMCC0401	**KT263446**	**KT263480**	**KT263515**	Capnodiales
A128 (TM)	*Lecanora bicincta*	*Sphaerellothecium atrinae*	A559	LMCC0170	**–**	**KT263478**	**KT263514**	Teratosphaeriaceae I
A72 (SAB)	*Lecanora polytropa*	*Carbonea supersparsa*	A554	LMCC0165	**KT263442**	**KT263475**	**KT263511**	Myriangiales
A94 (KGA)	*Lecanora intricata*	*Muellerella* - Li	A569	LMCC0180	**KT263444**	**KT263477**	**KT263512**	Myriangiales
A94 (DG)	*Lecanora intricata*	*Muellerella* - Li	A578	–	**KT263443**	**KT263476**	**KT263513**	Myriangiales
A56 (SAB)	*Lecanora intricata*	*Muellerella* - Li	A537	LMCC0155	**KT263430**	**KT263465**	**KT263499**	*Phoma* (Pleosporales)
A102 (DG)	*Acarospora fuscata*	*Polycoccum microstictum*	A558	LMCC0169	**KT263431**	**KT263466**	**KT263500**	*Phoma* (Pleosporales)
A160 (DG)	*Pertusaria lactea*	*Stigmidium eucline*	A542	LMCC0159	**KT263432**	**KT263467**	**KT263501**	*Phoma* (Pleosporales)
A254 (TM)	*Lecanora polytropa*	*Muellerella* - Lp	A593	LMCC0200	**KT263433**	**KT263448**	**KT263502**	*Phoma* (Pleosporales)
A23 (SAB)	*Lecanora intricata*	*Muellerella* - Li	A583	LMCC0190	**KT263438**	**KT263472**	**KT263508**	basal to Lichenotheliaceae
A56 (SAB)	*Lecanora intricata*	*Muellerella* - Li	A552	LMCC0164	**KT263436**	**KT263470**	**KT263506**	basal to Lichenotheliaceae
A100 (MY)	*Umbilicaria cylindrica*	*Stigmidium gyrophorarum*	A565	LMCC0176	**KT263441**	**KT263474**	**KT263510**	basal to Lichenotheliaceae
A100 (TM)	*Umbilicaria cylindrica*	*Stigmidium gyrophorarum*	A567	LMCC0178	**KT263439**	**KT263473**	**KT263509**	basal to Lichenotheliaceae
A128 (DG)	*Lecanora bicincta*	*Sphaerellothecium atrinae*	A595	LMCC0202	**KT263437**	**KT263471**	**KT263507**	basal to Lichenotheliaceae
A333 (KGA)	*Tephromel atra*	*Muellerella atricola*	A931	LMCC0272	**KT263435**	**KT263449**	**KT263505**	basal to Lichenotheliaceae
A440 (TM)	*Tephromel atra*	*Muellerella atricola*	A1057	LMCC0389	**KT263440**	**–**	**–**	basal to Lichenotheliaceae
A678 (SAB)	*Lecanora bicincta*	*Sphaerellothecium atrinae*	A977	–	**KT263434**	**–**	**KT263504**	basal to Lichenotheliaceae
A333 (LBM)	*Tephromel atra*	*Muellerella atricola*	A930	LMCC0305	**KT263424**	**KT263461**	**KT263495**	Lichenostigmatales
A651 (TM)	*Lecanora polytropa*	*Carbonea supersparsa*	A1039	LMCC0372	**KT263425**	**KT263462**	**KT263496**	Pleosporales
A675 (SAB)	*Tephromel atra*	*Taeniolella atricerebrina*	A1011	–	**KT263426**	**–**	**KT263497**	Pleosporales
A675 (MY)	*Tephromel atra*	*Taeniolella atricerebrina*	A1028	LMCC0350	**KT263427**	**KT263463**	**KT263498**	Pleosporales
A675 (MY)	*Tephromel atra*	*Taeniolella atricerebrina*	A1038	LMCC0371	**KT263429**	**KT263466**	**KT263503**	Pleosporales

### Culture isolation

A total of 130 lichen samples, comprising 25 different lichenicolous fungus-lichen host associations, were selected for culture isolations. Thallus areoles or lobes presenting lichenicolous fungal infections were removed with a sterile razor blade and put into an Eppendorf tube. The isolation protocol followed Yamamoto et al. (2002). The pieces, about 2 mm^2^, were washed three times for 15 minutes in distilled sterile water on a shaking bath, followed by a 30 minutes washing step with 500 μl of 1:10 dilution of Tween 80 to remove the possible external contaminations of bacteria and yeast (Bubrick and Galun 1986). A final washing step was carried out twice in distilled sterile water for 15 minutes. The clean fragments were dissected under the stereomicroscope using a sterile razor blade and single pieces were picked with a sterile needle, moistured with distilled sterile water, and transferred into agar tubes. In order to promote the growth of different fungi we inoculated the dissected fragments on six different media: *Trebouxia* Medium (TM, Ahmadjian 1967), Malt Yeast Extract Medium (MY, Ahmadjian 1967), Lilly and Barnett´s Medium (LBM, Lilly and Barnett 1951), Potato Glucose Agar (PGA; Sigma), Dichloran-Glycerol 18%-Agar (DG18; Sigma), Sabouraud-Agar (SAB; Sigma). Four tubes of the same medium were inoculated, resulting in a total of 24 tubes (inocula) for each original sample. The tubes were incubated in a growing chamber at 20 °C, with a light-dark regime of 14:10 hours with light intensity of 60-100 μmol photons m^-2^s^-1^ and 60% humidity. After three to five months, the inocula reached about 1-3 mm in diameter and it was possible to subculture and to prepare them for DNA extraction, sequencing and morphological analyses. The subcultures were set on agar plates using the same growth medium where the inoculum grew successfully. The cultured strains are deposited at the University of Graz in the culture collection of the first author LM and are preserved as cryostocks.

### Morphological analyses

Morphological and anatomical characters of the cultured strains were analysed using standard microscopic techniques and documented with photographs. Analyses and photographs were performed on 10 month to one year old subcultures considering the following characters: form of growth, branching of the hyphae and melanization. Small fragments of the mycelia were taken; squashed sections were mounted in water and studied by light microscopy. Images were acquired with a ZeissAxioCam MRc5 digital camera fitted to the microscope. Both images of growth habit and hyphae structure were digitally processed using the CombineZM software (www.hadleyweb.pwp.blueyonder.co.uk/). The photos were slightly refined in sharpness and color tone with Adobe Photoshop 7.0 and the figures were prepared with CorelDRAW X4.

### DNA extraction, amplification and sequencing

Small parts of the subcultured fungi were taken, transferred into 1.5 ml reaction tubes containing sterile beads for homogenization, frozen and ground using a TissueLyserII (Retsch). The DNA was then extracted following either the C-TAB protocol of Cubero et al. (1999) or using the DNeasy Plant Mini Kit (Qiagen, Austria). The industrial kit was used for those most melanized isolates for which the C-TAB protocol failed in extracting amplifiable DNA.

The identity of the cultured fungal strains was studied with sequences of the nuclear large and partial nuclear small ribosomal subunits (nucLSU and nucSSU) and the mitochondrial small ribosomal subunit (mtSSU). The nucLSU fragment was obtained in two pieces using primers SR6R (http://www.botany.duke.edu/fungi/mycolab) and LR5 for the upstream fragment, and LR3R and LR7 (Vilgalys and Hester 1990; http://www.biology.duke.edu/fungi/mycolab/primers.htm) for the downstream fragment. The nucSSU locus was amplified using the primers NS1 (White et al. 1990) and nuSSU0852 (Gargas and Taylor 1992). The mtSSU locus was amplified with primers mtSSU1KL (Lohtander et al. 2002) and MSU7 (Zhou and Stanosz 2001) or mtSSU1 and mtSSU3R (Zoller et al. 1999). PCRs amplifications were carried out with the Illustra™ puReTaq Ready-To-Go PCR Beads (GE Healthcare, UK Limited) with a reaction volume of 25μl and a primer concentration of 0,6 pmol/μl. The amplification of the genes followed touch-down PCR conditions as in previous studies (Muggia et al. 2011, 2013). PCR products were cleaned with E.Z.N.A.® Cycle Pure Kit (Omega Biotek, VWR) according to the manufacturer 's instructions. Both complementary strands were sequenced using the same PCR amplification primers by Microsynth (Sanger 3730xl from ABI, Vienna, Austria). Forward and reverse sequences were assembled into contigs and edited manually in BioEdit (Hall 1999).

### Alignment and phylogenetic analyses

We checked the identity of the newly generated sequences with sequences available in the GenBank database by blast similarity search (Altschul et al. 1990). Taxa which closest matched our sequences for a value not lower than 95% identity and the further closest related ones (up to 90% identity) were selected for the phylogenetic analyses. As our sequences showed closest matches with representatives of the classes Eurotiomycetes (particularly in the subclasses Chaetothyriomycetidae), Dothideomycetes, Leotiomycetes and Sordariomycetes, we prepared four different datasets representing each lineage (the multilocus sequences alignments are deposit at TreeBASE). We tried to include in each dataset the widest spectrum of taxon diversity by selecting, if possible, at least three taxa representatives of different families or orders of the four classes (Table S1, S2, S3). We based our selection also on previous phylogenetic analyses which considered the aforementioned classes (e.g. Zhang et al. 2006; Wang et al. 2006; Gueidan et al. 2008, 2011; Ruibal et al. 2009; Schoch et al. 2009; Huhndorf and Miller 2011; Untereiner et al. 2011; Muggia et al. 2013; Hyde et al. 2013; Maharachchikumbura et al. 2015; Suija et al. 2015, ). The datasets of Eurotiomycetes and Dothideomycetes were prepared in summer 2014 whereas those of Leotiomycetes and Sordariomycetes in January 2015. For this reason recent sequence data published subsequently summer 2014 by Gueidan et al. (2014) and Ertz and Diederich (2015) are not included here. For each dataset, outgroup taxa were chosen from the most closely related classes. Sequence alignments for each locus (nucLSU, nucSSU and mtSSU) and for each fungal class (Eurotiomycetes, Dothideomycetes, Leotiomycetes and Sordariomycetes,) were prepared manually in BioEdit (Hall 1999). Introns and ambiguous SNPs were removed from the alignment. For a number of specimens we were unable to generate sequences for all of the selected loci and for other taxa sequences were not available in GenBank. Therefore we present here a three-locus phylogenetic inference for the classes Eurotiomycetes and Dothideomycetes, and two-locus inferences for the classes Leotiomycetes and Sordariomycetes. The final phylogenetic analyses of the Eurothiomycetes dataset included a subset of the isolates, which were selected after having estimated a first phylogeny including all the isolates. As multiple isolates shared identical sequences, we selected for the final analyses as representatives those isolates obtained from different samples of the 25 lichenicolous fungus-lichen host associations which were grown on different media.

Combined data of different loci, either fully or partially congruent, have been commonly considered in phylogenetics (Dettman et al. 2003). We performed, therefore, as in previous studies (Kauff and Lutzoni 2002; Miadlikowska et al. 2006; Muggia Perez-Ortega et al. 2014), both single locus and combined datasets analyses. We analysed the single locus datasets with a Maximum Likelihood (ML) approach (Meson-Gamer and Kellogg 1996; Reeb et al. 2004) and the combined dataset using both maximum likelihood (ML) and Bayesian approaches. In both approaches the combined datasets were treated in partitions by genes nucLSU, nucSSU and mtSSU. The ML analyses were performed using the program RAxML v. 7.1.3 (Stamatakis et al. 2005). The GTRMIX model was applied both for the single loci and to each partition in the combined datasets (as only a single model of molecular evolution can be used across gene partitions in RAxML), and 1000 bootstrap replicates were run. The Bayesian Markov Chain Monte Carlo (B/MCMC) analyses were run in MrBayes 3.1.2 (Huelsenbeck and Ronquist 2003; Ronquist et al. 2005). The model of molecular evolution applied in the Bayesian analysis to each gene partition, the GTR+I+G model, was estimated in JModeltest v. 2.1.4 (Darriba et al. 2012) using the Akaike Information Criterion (Posada and Crandall 1998). The B/MCMC analyses were run with six chains simultaneously, each initiated with a random tree. Ten million generations for the Eurotiomycetes and Dothideomycetes datasets and five million generations for Leotiomycetes and Sordariomycetes datasets were run, respectively. Trees were sampled every 100 generations. The log-likelihood scores were plotted against generation time using Tracer 1.4 (Rambaut and Drummond 2007) to determine when the stationarity of likelihood values had been reached (e.g., the burn-in stage; Ronquist et al. 2005). Burn-in was set at half of the generations (the first 50,000 and 25,000 sampled trees for the two datasets groups respectively) and the majority rule consensus trees were calculated from the posterior samples of 50,001 and 25,001 trees, respectively. The convergence of the chains was confirmed by the convergent diagnostic of the Potential Scale Reduction Factor (PSRF), which approached 1 (Ronquist et al. 2005). The phylogenetic trees were visualized in TreeView (Page 1996).

## Results

### Culture isolation

A total of 248 fungal cultures from 77 host lichen thalli were isolated and identified to date: 191 belong to the subclass Chaetothyriomycetidae, 36 to the class Dothideomycetes, 12 to Leotiomycetes and 9 to Sordariomycetes. We obtained 21 additional isolates that corresponded to the lichen mycobionts (not shown). The majority of the strains, 24%, grew on TM, 22% grew on LBM, 20% on SAB, 16% on KGA, 13% on MY and 5% were isolated on DG media. Cultured mycobionts represented 2% of the grown isolates. From these cultures we obtained in total 710 new sequences: 244 for nucLSU rRNA gene, 237 for nucSSU rRNA gene and 229 for mtSSU rRNA gene (Table 1, Table 2, Table S3). The diversity of fungi isolated from lichen thalli, and which did not represent the mycobiont of the lichen symbiosis, varied among the 77 original thalli. The specificity of the isolated fungi neither correlates with the presence of any observed lichenicolous fungus nor with the identity of the lichen mycobiont. Fungi belonging to the same lineage were isolated from multiple thalli representing the same association of lichen and lichenicolous fungus, but also from the same lichen host species infected by different lichenicolous fungi (from hosts not growing in vicinity) and from other different associations of lichen and lichenicolous fungus (Tables 1, 2, S3). For example, we isolated up to five different lineages of fungi in two lichen individuals; fungi of four different lineages were isolated from only a single thallus, fungi of three different lineages were isolated from nine thalli. Fungi representing two different lineages were retrieved from 21 thalli, and fungi representing one lineage were obtained from 40 thalli.

### Phylogenetic and morphological analysis of Chaetothyriomycetidae (Fig. 1, Fig. 2, Table 1 and Table S1)

**Fig. 1:**
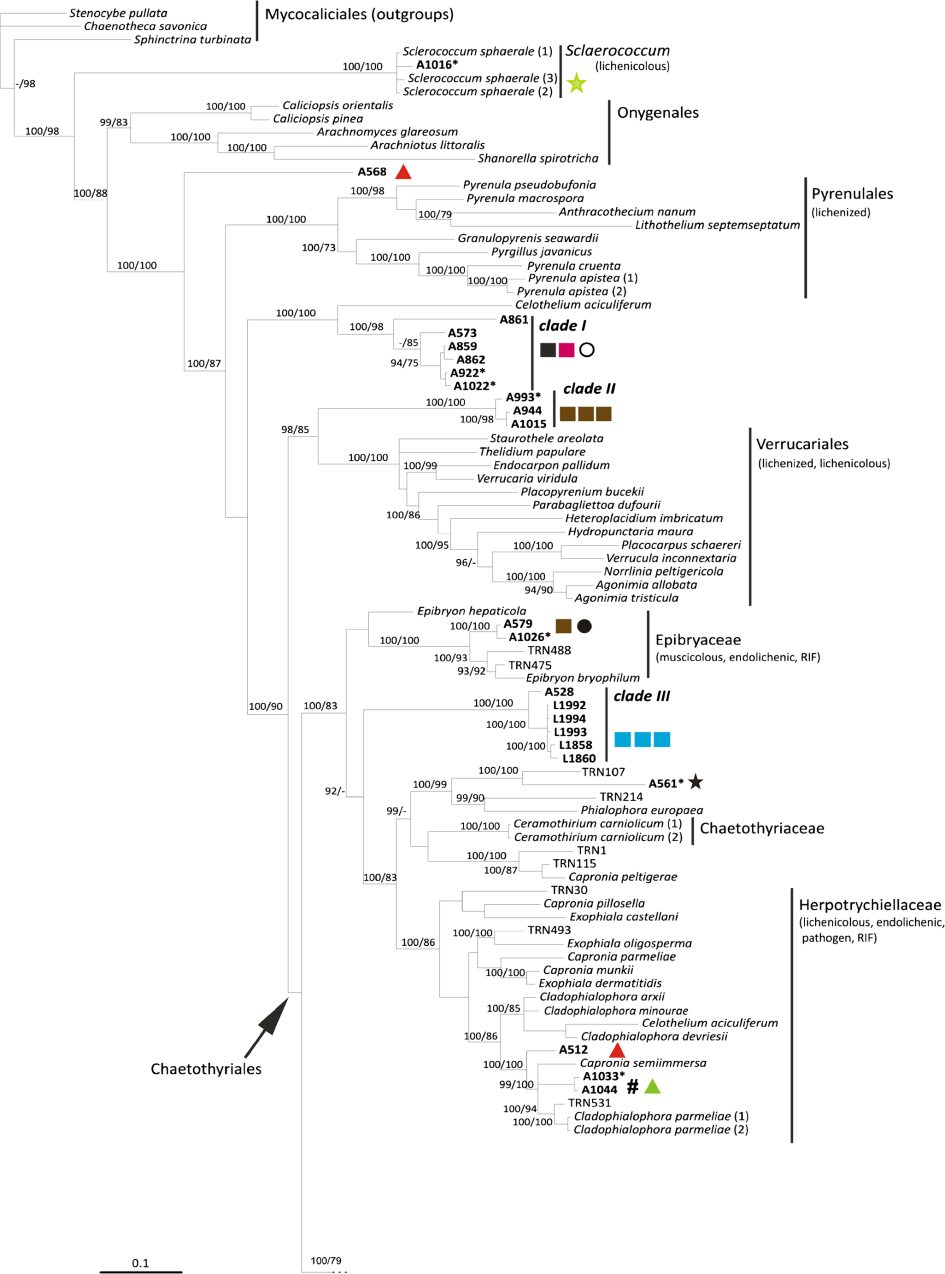

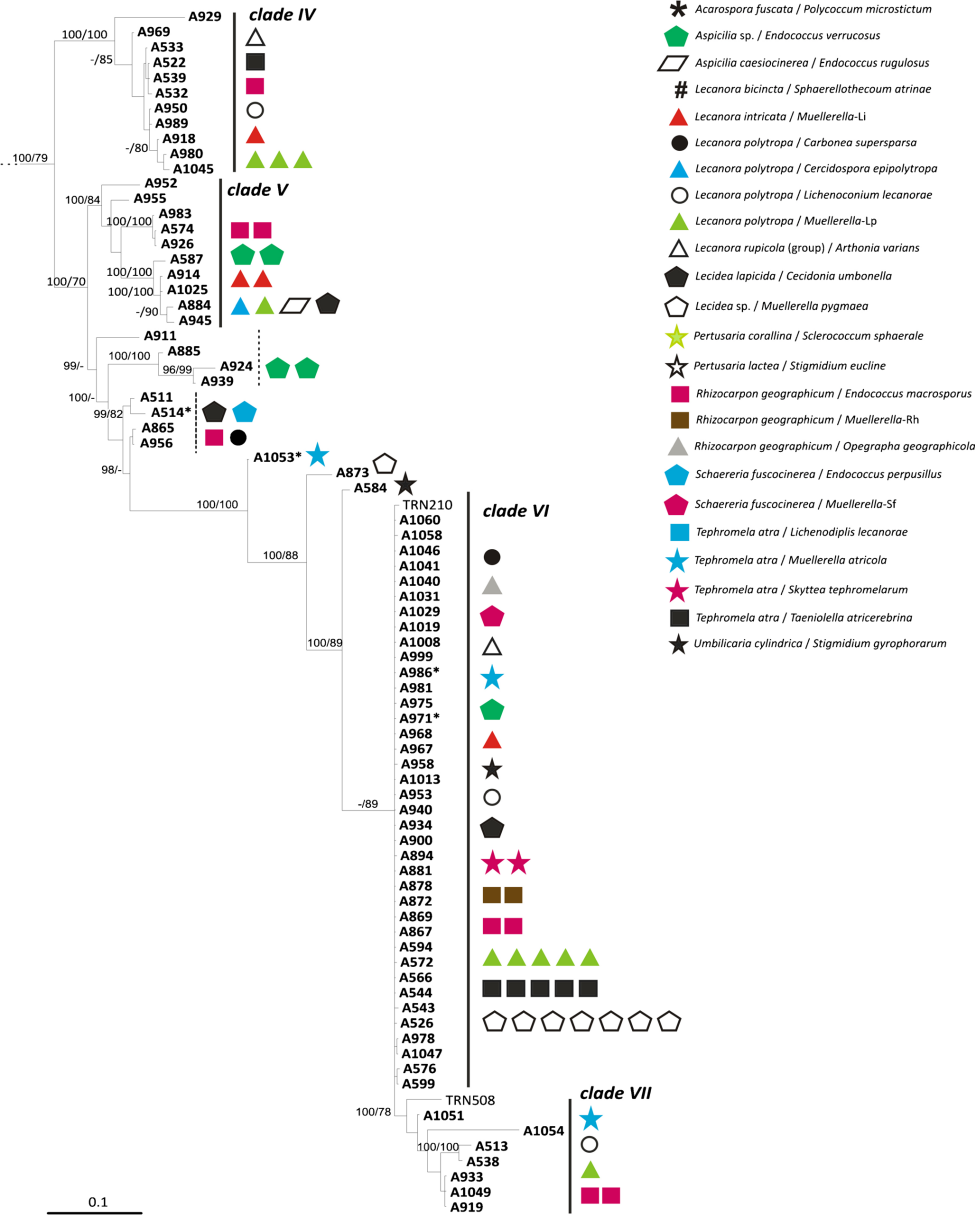
Multilocus phylogenetic inference of Eurotiomycetes. The ML and the Bayesian phylogenetic hypotheses were inferred from the combined dataset of nucLSU, nucSSU and mtSSU loci and corresponded in their topologies; the ML analysis is shown. Bayesian posterior probabilities (PP ≥ 95 %) and ML bootstrap support values (≥ 70 %) are reported above branches (PP/bootstrap value). Newly identified clades of isolated fungi obtained from this study are highlighted in bold and are labelled as *clade I* to *VII*. Symbols indicate the different lichen host-lichenicolous fungal associations which represent the original thallus from where the fungal strains were isolated. A symbol reported multiple times for a clade indicates the number of different original thalli sharing the same lichen host-lichenicolous fungal association. Fungal life-styles are reported in parenthesis. Samples labelled with an asterisk (*) are those photographed in Fig. 2

**Fig. 2:**
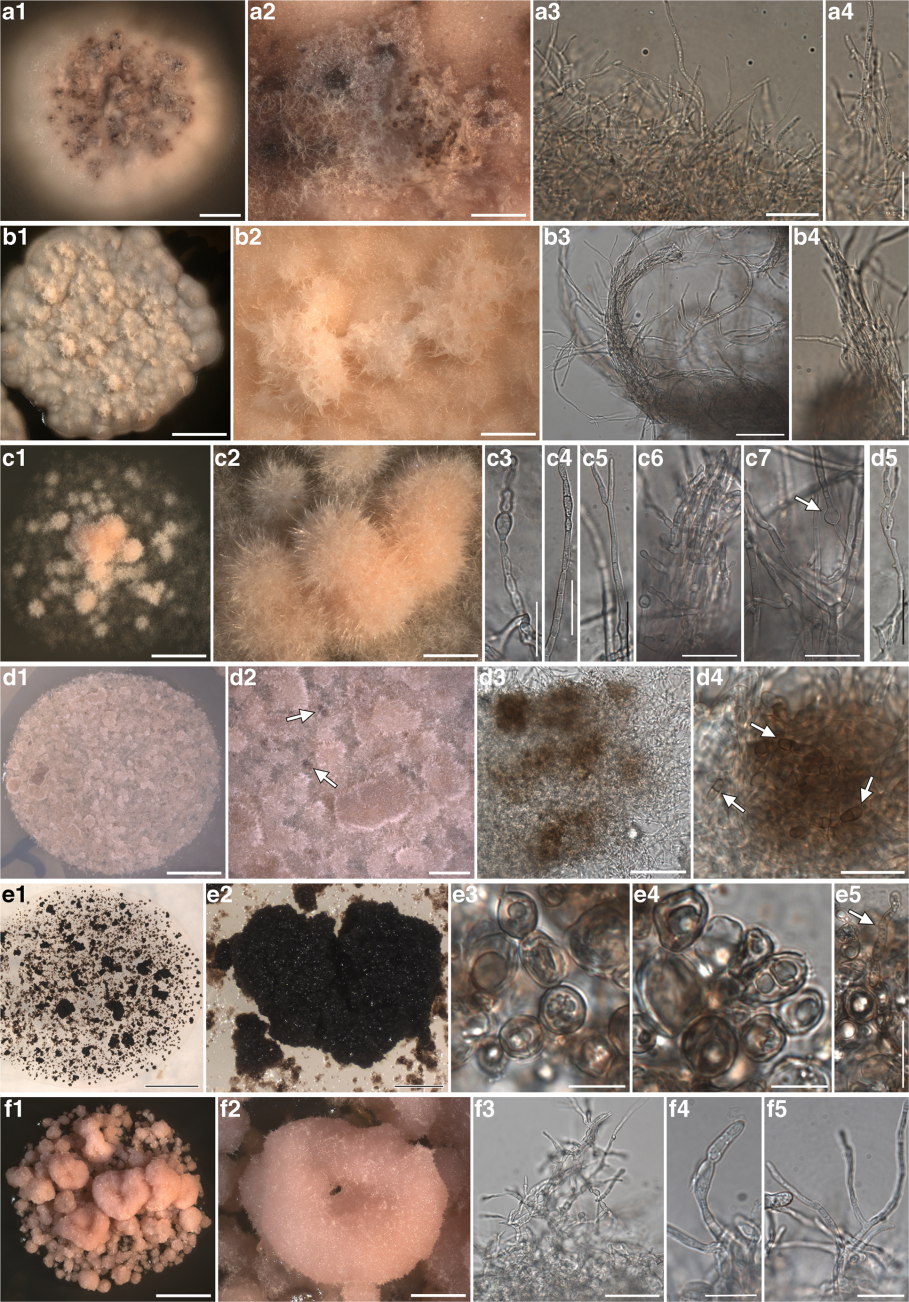

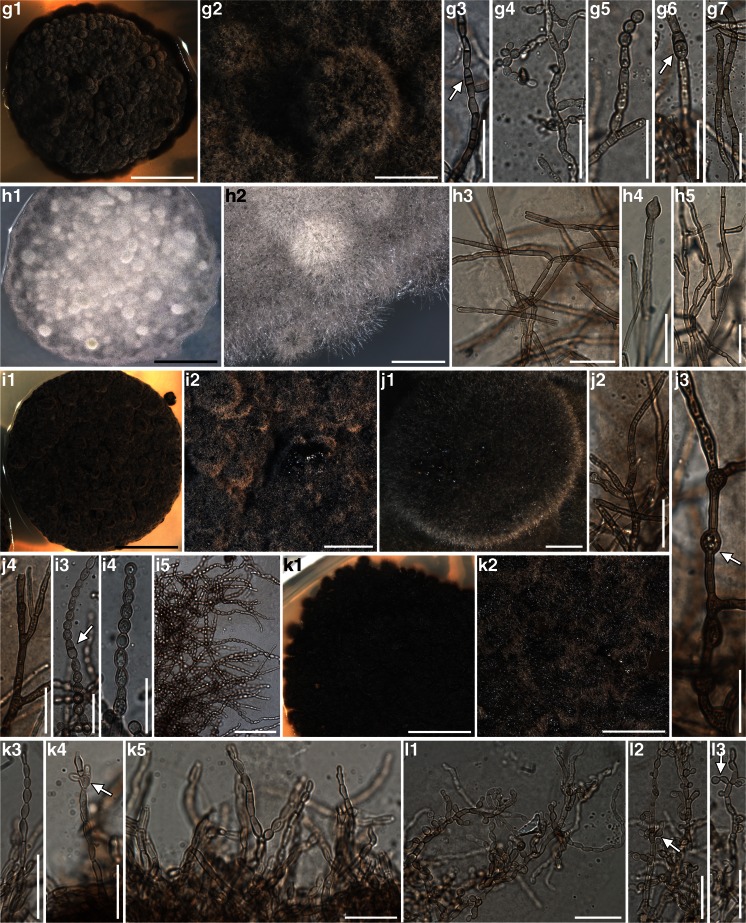
Habitus of one year old, representative, cultured fungal strains belonging to Eurotiomycetes and included in the phylogenetic analysis of Fig. 1. Anatomical structures were photographed from squashed sections mounted in water. Samples are reported with their number and the clade to which they belong as in Fig. 1. A1-A4) A922 (*clade I*) - A1, A2 habitus of the mycelium; A3, A4 fine, hyaline hyphae. B1-B4) A1022 (*clade I*) - B1, B2 habitus of the mycelium; B3, B4 fine, hyaline hyphae, gathering in entangled, plectenchymatous strands. C1-C7) A993 (*clade II*) - C1, C2 habitus of the mycelium; C3-C7 hyaline hyphae with branching and globose cells intercalating with cylindrical cells. D1-D5) A528 (*clade III*) - D1, D2 habitus of the mycelium; D3, D4 brown cell structures containing conidia-like cells (arrow in D4); D5, hyaline hyphae. E1-E5) A1026 (Epibryaceae) - E1, E2 habitus of the mycelium; E3-E5 dense aggregate of roundish, melanised cells containing inclusions, filamentous hyphae rarely present (E5). F1-F5) A1016 (*Sclerococcum*) – F1, F2 habitus of the mycelium; F3-F5 hyaline hyphae with branching and cylindrical, more or less elongated cells. G1-G7) A1053 (single branch, basal to *clade VI*) – G1, G2 habit of the melanized mycelium; G3-G7 hyphae composed by melanised, single or 1-septate cells, with numerous apical and lateral buds. H1-H5) A514 (basal to *clade VI*) – H1, H2 habitus of the mycelium; H3-H5 melanized hyphae with cylindrical cells, apical bud with roundish cells (H4), infrequent branching. I1-I5) A986 (*clade VI*) – I1, I2 habitus of the melanized mycelium; I3-I5 hyphae composed by globose, roundish cells, sometimes 1-septate (I3 arrow), with thick cell wall. J1-J4) A971 (*clade VI*) – J1 habitus of the melanized mycelium; J2-J4 melanized hyphae with cylindrical cells intercalating with roundish cells (J3 arrow), ramifications originate both from the cylindrical and the roundish cells. K1-K5) A1033 (Herpotrichiellaceae) – K1, K2 habitus of the melanized mycelium; K3-K5 melanized hyphae composed by elliptical, subcylindrical and subglobose cells constricted at the septa. L1-L3) A561 (basal to Chaetothyriaceae) melanized hyphae composed by elliptical, subcylindrical and subglobose cells constricted at the septa, frequently laterally and apically budding. Scale bars =4 mm (D1, G1, H1, I1, K1), 3 mm (A1, B1, C1, E1, F1), 1 mm (B2, D2, I2, K2), 0.5 mm (A2, C2, E2, F2, G2, H2, J1), 50 μm (B3, D3, F3, I5), 20 μm (A3, A4, B4, C3-C7, D4, D5, F4, F5, G3-G7, H3-H5, I3, I4, J2-J4, K3, K4, L2, L3)

The phylogenetic relationships recovered in Chaetothyriomycetidae are highly congruent with previous studies of Gueidan et al. (Gueidan et al. 2008, Gueidan et al. 2011) and Diederich et al. (2013). There were no significant incongruences between single locus (not shown) and multilocus trees. The only exception is the clade of *Sclerococcum sphaerale*, which is placed in our multilocus reconstruction at the base of Chaetothyriomycetidae, possibly due to the availability of only the nucLSU marker (Fig. 1). In this *Sclerococcum sphaerale* clade we recovered the single isolate A1016. A1016 was isolated from a thallus of *Pertusaria corallina* infected by *Sclerococcum sphaerale*, and this placement seems to confirm the identity of the lichenicolous fungus. This isolated strain forms pale pinkish, compact mycelia with thin, hyaline hyphae (Fig. 2 F1-F5). *Clade I* is represented by six isolates (from three host species), which together with *Celothelium cinchonarum* are basal to the split between Verrucariales and Chaetothyriales. These isolates are similar in morphology, forming white mycelia composed by thin, hyaline hyphae, which occasionally gather in thick, plectenchymatous strands (Fig. 2 A1-A4, B1-B4). *Clade II* is represented by three isolates: they come from three different thalli of the same lichen host- lichenicolous fungus association (*Rhizocarpon geographicum* – *Muellerella pygmaea-*Rh). These strains also present a pale pinkish mycelium, but hyphae are formed by cylindrical to semi-elliptical cells which are occasionally intercalated by roundish cells (Fig. 2 C1-C7). Two samples, A579 and A1026, are nested within Epibryaceae, the lineage formed by *Epibryon* and two rock-inhabiting fungi. The mycelium of these isolates is a dense aggregate of roundish, melanised cells containing inclusions, and filamentous hyphae are rarely present (Fig. 2 E1-E5). *Clade III* represents the lichenicolous fungus *Lichenodiplis lecanorae* (Muggia et al. in prep.), which appears here basal to the split between the families Epibryaceae, Chaetothyriaceae and Herpotrichiellaceae. The identity of these isolates is also confirmed by the conidiomata-like structures and the conidia that are observed in the cultures (Fig. 2 D1-D4). Herpotrichiellaceae is here the most represented family of Chaetothyriales and comprises ecologically diverse fungi including human pathogens (*Exophiala dermatitidis* and *Capronia semiimmersa*), lichenicolous fungi (*Capronia peltigerae* and *Cladophialophora parmeliae*) and rock inhabiting fungi (Gueidan et al. 2008; Gueidan et al. 2011, Gueidan et al. 2014). Four newly cultured isolates are nested in this main Chaetothyriales lineage. A561 is nested in a clade with RIF and *Phialophora europaea*, and is morphologically similar to other previously isolated black RIFs (Fig. 2 L1-L3), having melanized hyphae frequently budding laterally and apically. Three other samples are nested in a clade with *Cladophialophora parmeliae* and *Capronia semiimmersa*. The isolates are characterized by melanized mycelia, with branching hyphae composed by elliptical, subcylindrical and subglobose cells constricted at the septa (Fig. 2 K1-K5).

In Chaetothyriales, the majority of the isolates group into subclades of a fully supported lineage sister to Chaetothyriales. Within this lineage we distinguished the main *clades IV*, *V*, *VI* and *VII* (as subclade of *clade VI*, Fig. 1), each represented by more than four isolates. The other isolates are placed on separate smaller clades in this large assemblage of branches*. Clade IV* and *clade V* include isolates from six and seven, respectively, different lichen host- lichenicolous fungus associations. *Clade VI* contains the majority of the isolates which come from 16 different lichen host - lichenicolous fungus associations. These include lichens infected by known but unrelated lichenicolous fungi. With the exception of few isolates, such as A514 (Fig. 2 H1-H5) and A511, which lack melanized mycelium, all the fungal strains included in this big assemblage of lineages are characterized by melanized mycelia. However, two main morphologies are observed among the strains: *i*) mycelia with filamentous, branching hyphae composed by cylindrical cells, usually aseptate (rarely 1-septate), intercalating by roundish cells, *ii*) mycelia with hyphae composed exclusively by globose, roundish cells, sometimes 1-septate, forming dense assemblages and budding.

Except for the *Sclerococcum* clade and the *clade III*, we do not find clear evidence of correspondence of certain lineages with other lichenicolous fungi infecting the lichen samples.

### Phylogenetic and morphological analysis of Dothideomycetes – (Fig. 3, Fig. 4, Table 2 and Table S2)

**Fig. 3:**
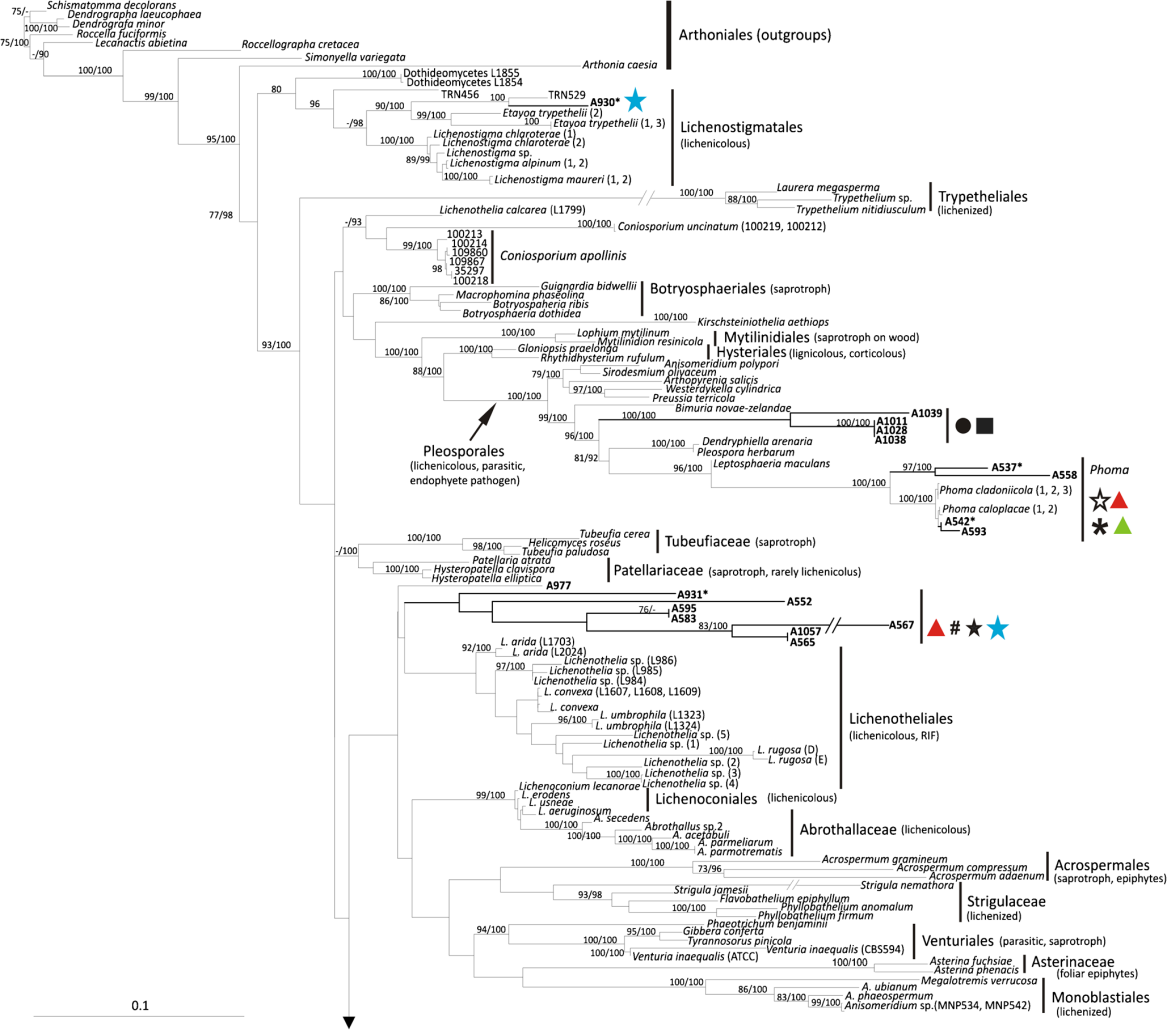

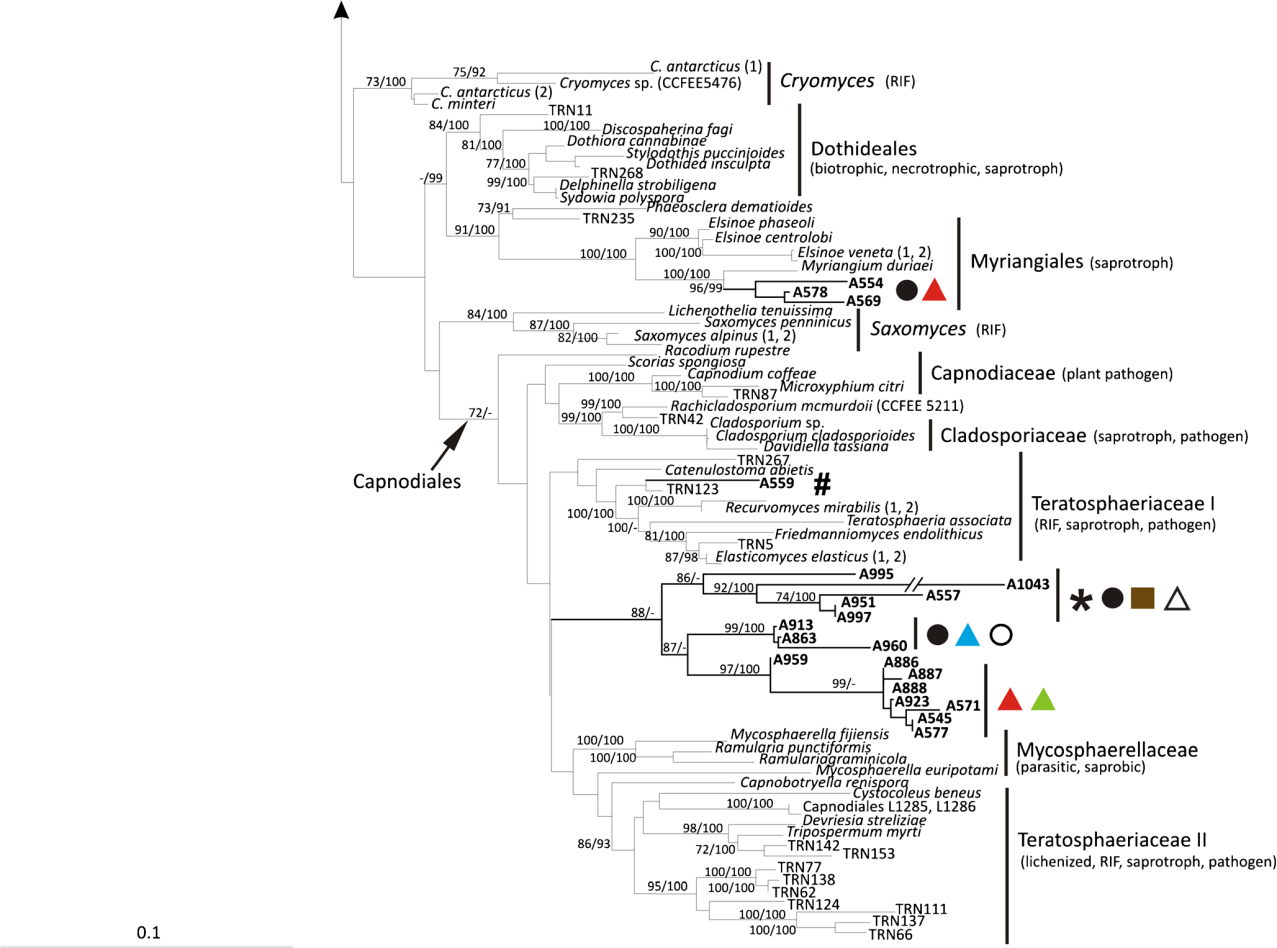
Multilocus phylogenetic inference of Dothideomycetes. The ML and the Bayesian phylogenetic hypotheses were inferred from the combined dataset of nucLSU, nucSSU and mtSSU loci and corresponded in their topologies; the ML analysis is shown. ML bootstrap support values (≥ 70 %) and Bayesian posterior probabilities (PP ≥ 95 %) are reported above branches (bootstrap value/PP). Fungal isolates obtained from this study are highlighted in bold. Symbols indicate the different lichen host-lichenicolous fungal associations as reported in Fig. 1. Fungal life-styles are reported in parenthesis. Samples labelled with an asterisk (*) are those photographed in Fig. 4

The phylogenetic relationships recovered in Dothideomycetes are highly congruent with previous studies of Ruibal et al. (2009), Lawrey et al. (Lawrey et al. 2011) Lawrey Diederich et al. 2012, Muggia et al. (2013); Hyde et al. (2013). Topological congruence was recovered between the Bayesian and the maximum likelihood analyses and among the single locus analyses. Also in Dothideomycetes the isolates are nested in clades together with fungi of diverse ecological niches and presenting different lifestyles (Fig. 3).

**Fig. 4:**
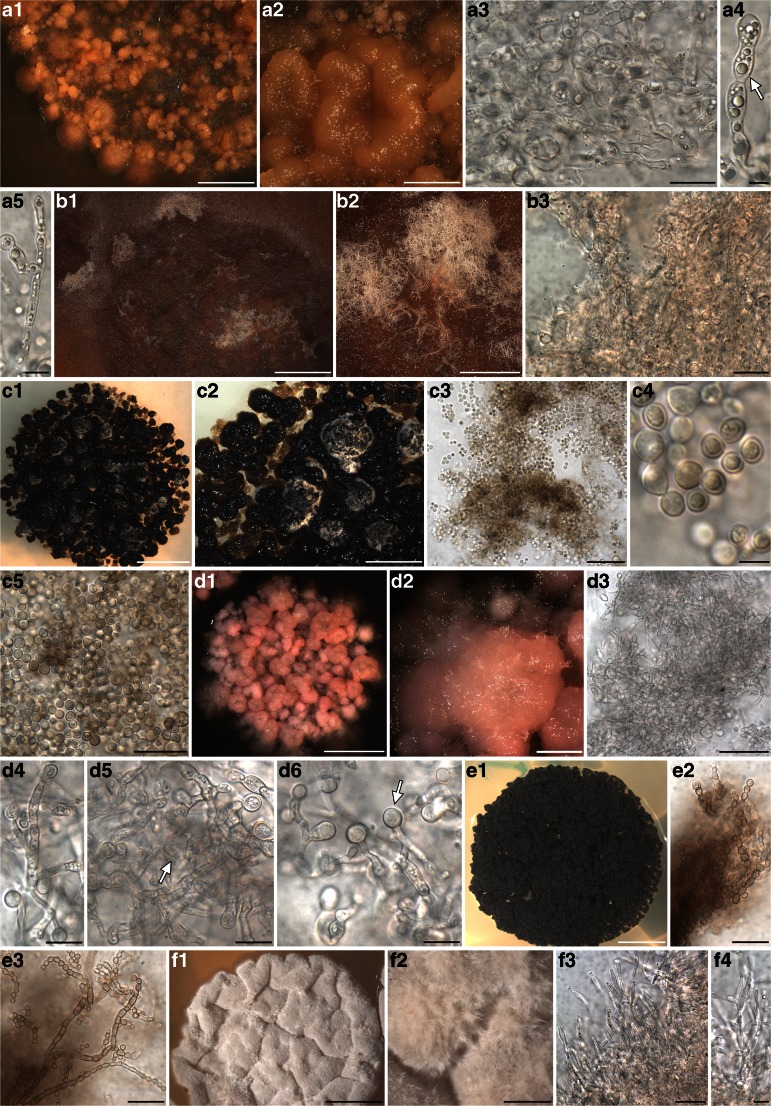

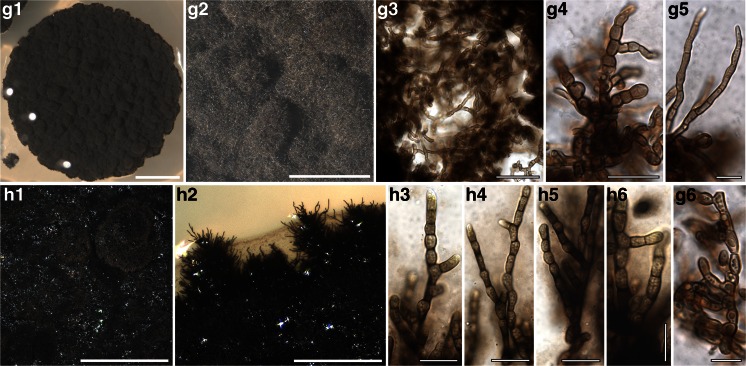
Habitus of one year old, representative, cultured fungal strains belonging to Dothideomycetes and included in the phylogenetic analysis of Fig. 3. Anatomical structures were photographed from squashed sections mounted in water. Samples are reported with their number and the clade to which they belong as in Fig. 3. A1-A5) A537 (*Phoma*) – A1, A2 habitus of the mycelium; A3-A5 hyaline hyphae with inclusions forming a dense aggregate. B1-B3) A542 (*Phoma*) – B1, B2 habitus of the mycelium, B3 dense aggregate of hyphae. C1-C5) A930 (Lichenostigmatales) – C1, C2 habitus of the mycelium; C3-C5 yeast-like melanised cells forming dense aggregates. D1-D6) A931 (clade sister to Lichenotheliales) – D1, D2 habitus of the mycelium; D3-D6 plectenchymatous structure of hyaline hyphae with cylindrical cells, round cells as buds at the apexes of the hyphae (arrows). E1-E3) A 567 (clade sister to Lichenotheliales) – E1 habitus of the mycelium; E2, E3 melanized hyphae composed by elliptical and subcylindrical cells constricted at the septa, laterally budding. F1-F4) A554 (Myriangiales) – F1, F2 habitus of the mycelium; F3, F4 thin, hyaline hyphae. G1-G6) A559 (Teratospaheriaceae I) – G1, G2 habitus of the mycelium; G3-G6 melanized hyphae, branching and composed by cylindrical to subglobose cells. H1-H6) A960 (clade nested in Teratosphaeriaceae) H1, H2 habitus of the mycelium, hyphae develop inside the growth medium; H3-H6 melanized hyphae, branching and composed by cylindrical to subglobose cells. Scale bars =4 mm (A1, C1, D1, E1, F1, G1), 3 mm (B1), 2 mm (B2, H1), 1.5 mm (C2), 1 mm (A2, D2, F2, G2), 0.4 mm (H2), 50 μm (G3), 40 μm (C3, D3), 20 μm (A3, C5, E2, E3, F3, G4, H3-H6), 10 μm (A5, B3, D4-D6, F4, G5, G6)

The isolate A930 is recovered within Lichenostigmatales, which includes lichenicolous fungi and RIFs. A930 is morphologically identical to the *Lichenostigma* cultures isolated by Ertz et al. (2014), presenting yeast-like, budding, melanized cells. Four isolates form a fully supported clade nested in Pleosporales. Also in Pleosporales, four further isolates group together with lichenicolous species of the genus *Phoma*; however they were isolated from thalli of four different lichen host-lichenicolous fungi associations and none of them showed the symptomatic presence of *Phoma* species*.* These isolates form whitish to pale pinkish mycelia, composed by hyaline hyphae distributed to form a dense aggregate (Fig. 4 A1-A5, B1-B3). Seven isolates represent a lineage sister to Lichenotheliales; these isolates originate also from four thalli representing different lichen hosts infected by different lichenicolous fungi. The isolates comprise both melanized and non-melanized fungi (Fig. 4 D1-D6 and E1-E3). Three isolates are recovered in Myriangiales, a lineage of saprobic fungi; they present white mycelium of very thin hyaline hyphae (Fig. 4 F1-F4). The single isolate A559 is recovered as a member of Teratosphaeriaceae I. The remaining isolates group as a single lineage in Capnodiales, being nested among the clades Teratosphaeriaceae I, Teratosphaeriaceae II and Mycosphaerellaceae. In this lineage we identify three subclades, even though all isolates have a similar morphology, with dark, melanized mycelia composed by suglobose to cylindrical cells with rough cell wall and sometimes constricted at the septa (Fig. 4 G1-G6, H1-H6).

### Phylogenetic and morphological analysis of Leotiomycetes and Sordariomycetes (Fig. S1, Fig. S2, Table S3 and Table S4)

Only 15 and nine isolates have been identified as Leotiomycetes (Helotiales) and Sordariomycetes, respectively. Within Leotiomycetes none of our isolates is closely related to the lineage Encoelioideae, where recently lichenicolous fungi were identified to belong (Suija et al. 2015). Five isolates are placed with unresolved position at the base of Leotiomycetes; one isolate is closely related to *Leotia lubrica* (saprotroph among mosses and plant rests, Kuo 2003) and *Microglossum olivaceum* (a grassland species, Fleming 2001). Eight isolates obtained from three different combinations of lichen host and lichenicolous fungus are closely related to *Mitrula paludosa* (a species known from swamps and bogs, Wang et al. 2005).

Three isolates are identified in Xylariales within the Sordariomycetes, one isolate is nested in Hypocreales (including insect parasitic species, mycoparasites, endophytes and saprotroph, Gazis et al. 2014), and five isolates, deriving from three different lichen host-lichenicolous fungi associations are recovered in Coniochaetales (saprotrophs, leaf and root endophytes, plant pathogens, Zhang et al. 2006). Strains of both Leotiomycetes and Sordariomycetes form pale pinkish to white mycelia (Fig. S3); melanization was seldom observed and was restricted only to localized parts of the culture (Fig S3 G).

## Discussion

Rock-inhabiting alpine lichens are exposed to harsh environmental conditions, with drastic and sometimes sudden changes in temperature and hydration, as well as UV radiation. Conceivably, only fungi that tolerate such fluctuating conditions can persist or grow in lichens. In addition, these fungi must cope with the diverse and highly concentrated extracellular secondary products of their host species. We already found a surprising number of lichenicolous fungi in lichens (Fleischhacker et al. 2015), and evidence for a high number of additional, cryptically occurring fungi. Here we provided a comprehensive set of isolates of the culturable fungal fraction in lichens from an alpine habitat for a survey of their phylogenetic relationships, with special emphasis on members of Dothideomycetes and Chaetothyriomycetes.

Molecular data and the morphological analyses seem to confirm the identity of only two symptomatic lichenicolous fungal species with Eurotiomycetes. The isolates obtained from thalli infected by *Sclerococcum sphaerale* indeed group within the lineage *Sclerococcum* (Diederich et al. 2013). The formation of conidiomata and conidocells was observed in multiple cultured fungi from different thalli with infections of *Lichenodiplis lecanorae*. This proved the identity of the culture with the original infection of the lichenicolous hyphomycete.

Except for the above mentioned clades, we do not find clear evidence of correspondence of certain lineages with other lichenicolous fungal species infecting the lichen samples. Some of the observed lichenicolous fungi cannot be the origin of the sequenced cultures, since these belong to completely unrelated groups (e.g. *Arthonia*, *Carbonea*, *Cecidonia*, *Opegrapha*, *Skyttea*, *Stigmidium*; Ertz et al. 2009, 2014; Schmull et al. 2011; Suija et al. 2015). It is likely that the *clade IV* and *V*, and the plenty of clades with few representatives, so far correspond to lineages of still unknown fungi which may occur widespread in lichen thalli from rocks, but are unapparent to the eye.

The present phylogenetic results also show that some of the detected fungi are closely related to lichenicolous fungi as well as to fungi known from diverse other ecological niches. Two isolates are closely related to the genus *Epibryon*, which was originally described as bryophilous (Döbbeler 1978). It is now emended by non-lichenized lichenicolous species (Zhurbenko and Hafellner 1999; Sérusiaux et al. 1999), which demonstrates cross-kingdom host switches in this monophyletic genus. The host lichens of the *Epibryon* strain were also visibly infected by the genera *Carbonea* and *Muellerella*, respectively. Also *Muellerella* comprises species on bryophytes and lichens (Döbbeler and Triebel 1985), but its relationship with *Epibryon* requires further study. Clearly *Carbonea*, as a member of Lecanoraceae, is unrelated. The results suggest that *Epibryon* could occur also as a non-symptomatic lichen inhabitant, which agrees with the previous results of U’Ren et al. (2010), who discovered a group of fungi capable to live cryptically in both lichens and mosses. The cryptic presence of otherwise symptomatic lichenicolous fungi is also demonstrated by isolates placed with the lichenicolous lineage of the anamorph genus *Phoma*, and those strains which are nested with lichenicolous species of *Capronia* and *Cladophialophora*. None of these isolates, however, originated from thalli which were visibly infected by either *Phoma*, *Capronia* or *Cladophialopora*. The high similarity (>95%) that the new sequences showed with the already available *Phoma* sequences suggests that the isolated strain could represent closely related *Phoma* species.

The majority of the isolates are melanized fungi, which closely resemble previously studied rock-inhabiting fungi (RIF) and in fact are closely related to them. The presence or absence of these fungi in hosts of the same area seems to be largely unpredictable, unspecific and facultative. Rather than indicating host specificity, they seem to be broadly tolerant species whose presence might depend more on physical parameters. Nonetheless, lineage *clade VI* (Chaetothyriales) seems to be rather ubiquitous in lichens. All selected fungi likely represent the same species occurring in many thalli and in combination with different lichenicolous fungi.

The finding of few isolates in Myriangiales, Xylariales, Hypocreales and Coniochaetales is quite interesting, as this is the first record for lichens from rocks; members of these groups are mainly biotrophic plant-associated fungi, endophytes, saprotrophs on wood and insect parasites. Fungi in Xylariales were, though, already isolated from lichen thalli from other ecological niches (Ding et al. 2009, U’Ren et al. 2012). However, no diagnostic structure hinting at these fungi have ever been observed under the microscope. Arnold et al. (2009) first suggested that fungi may live a symptomless life in lichens and coined the term ´endolichenic fungi´ for such organisms. Arnold and co-authors (Arnold et al. 2009, U’Ren et al. 2010, 2012) also have studied lichens from different habitats, such as tropical forest, temperate, boreal and arctic locations. Though some of these lichens are of the same mycobiont genera as the species included in this study, Arnold and colleagues found a higher proportion of fungi in Leotiomycetes and Sordariomycetes, more closely related to lineages of plant endophytes, rather than to the lineages predominantly found in this survey. It is likely that the taxonomic diversities recovered between the two surveys correlates with the local vegetation and geologic histories of the regions. The cryptic occurrence of fungi has been also found in different environments (Stergiopoulos and Gordon 2014), and even included plant pathogens (Malcolm et al. 2013).

We also isolated fungi which constitute two monophyletic lineages, both closely related to orders and families of lichenicolous and lichenized genera, RIF and pathogens in Dothideomycetes: the first closely related to Lichenotheliales, the second nested in Capnodiales. The first lineage is closely related to species of the genus *Lichenothelia*, which are known to share multiple lifestyles on rocks (Hyde et al. 2013; Muggia et al. 2013, 2015). They dwell on bare rock surfaces, but are often found associated with free living algae also present on the rocks. Some species specialize as lichen parasites and seem to associate with the lichen photobiont (Muggia et al. 2015). Some oligotrophic fungi apparently improve their carbon supply by attaching to microscopic algae. A direct involvement of black fungi in fungal-algal interactions was earlier described as a balanced algal parasitism (Turian 1977). Several rock-inhabiting and lichen-inhabiting microcolonial fungi develop into lichenoid structures within months when co-cultured with algae obtained from lichen thalli (Gorbushina et al. 2005; Brunauer et al. 2007). Gorbushina and Broughton (2009) showed an example with a co-culture of *Nostoc* and a rock-inhabiting fungus (*Sarcinomyces*). They observed a specific spatial arrangement of both organisms and growth alterations in the photosynthetic cyanobacteria suggested a specific interaction. Therefore black fungi that loosely associate with algae in nature might be interpreted as “lichenoids” and are considered prime forms of symbiosis (Muggia et al. 2013).

The apparent ability of black fungi to associate loosely with algae sheds an interesting light on the evolution of lichens. In fact some of the rock-inhabitants are basal to the large lichenized Ascomycete lineages Arthoniomycetes and Verrucariales (Gueidan et al. 2008; Ruibal et al. 2009). Otherwise, the lichenized life styles are scattered in various clades of Dothideomycetes (Muggia et al. 2008; Ruibal et al. 2009; Nelsen et al. 2009), where lichen thallus morphology remains generally simple. However, not all of the lineages do associate with algae or establish lichen symbioses. Some Dothideales have evolved into highly adaptable and versatile species -e.g. *Aureobasidium pullulans* commonly found on leaf surfaces of plant- but have not been found to be associated with lichens.

In our survey, fungi of unrelated lineages were recovered several times from individual lichen thalli. This may indicate that there is no competition between the different fungi, which complies with a concept of niche-sharing (Crous et al. 2009), and that the occurrence of certain lineages does not implicate the presence or absence of others. Lichen-associated fungi, which do not develop any diagnostic structure on the thallus host, use the host just for their own cryptic internal life, likely awaiting the most suitable substrate/host to propagate. Perhaps not all isolated fungi grow equally well in lichens, and we cannot exclude that some might be present as spores or small germlings, while others form mycelia networks in their hosts. We often see mycelia of melanized fungi on the lichens and expect their growth is well adapted to the poikilohydric lichen habitat. The symbiotic structures of the lichen thalli function as a shared habitat of phylogenetically diverse stress-tolerant fungi, some of which use their host as protection, while others use it as nutrition sources in otherwise hostile environments.

## Electronic supplementary material

Figure S1. Multilocus phylogenetic inference of Leotiomycetes. The ML and the Bayesian phylogenetic hypotheses were inferred from the combined dataset of nucLSU and nucSSU loci and corresponded in their topologies; the ML analysis is shown. ML bootstrap support values (≥ 70%) and Bayesian posterior probabilities (PP ≥ 95%) are reported above branches (bootstrap value/PP). Fungal isolates obtained from this study are highlighted in bold. Symbols indicate the different lichen host-lichenicolous fungal associations as reported in the legend. Samples labelled with an asterisk (*) are those photographed in Fig. S3. (JPEG 2367 kb)

Figure S2. Multilocus phylogenetic inference of Sordariomycetes. The ML and the Bayesian phylogenetic hypotheses were inferred from the combined dataset of nucLSU and nucSSU loci and corresponded in their topologies; the ML analysis is shown. ML bootstrap support values (> 70%) and Bayesian posterior probabilities (PP > 95%) are reported above branches (bootstrap value/PP). Fungal isolates obtained from this study are highlighted in bold. Symbols indicate the different lichen host-lichenicolous fungal associations as reported in the legend. Fungal life-styles are reported in parenthesis. Samples labelled with an asterisk (*) are those photographed in Fig. S3. (JPEG 2947 kb)

Figure S3. Habitus of one year old, representative, cultured fungal strains belonging to Leotiomycetes and Sordariomycetes and included in the phylogenetic analysis of Fig. S1 and Fig. S2 respectively. Leotiomycetes: A) A899, B, C) A907, D) A910, E, F) A935. Sordariomycetes: G) A592 (Xylariales), H, I) A560 (Hypocreales), J) A524 (Coniochaetales), K, L) A890 (Coniochaetales). Sclae bars = 4 mm (A, B, D, F, G, H, J, K), 2 mm (C, I), 1 mm (E), 0.5 mm (L). (JPEG 8509 kb)

Table S1. List of Eurotiomycetes taxa retrieved from GenBank and selected for the phylogenetic analysis of Fig. 1. ID (if available) and NCBI accession numbers are reported. Outgroups are labelled by an asterisk. (DOCX 17 kb)

Table S2. List of Dothideomycetes taxa retrieved from GenBank and selected for the phylogenetic analysis of Fig. 2. ID (if available) and NCBI accession numbers are reported. Outgroups are labelled by an asterisk. (DOCX 28 kb)

Table S3. List of Leotiomycetes and Sordariomycetes isolates obtained in this study and included in the analyses of Fig. S1 and Fig. S2 respectively. Number of the original lichen thallus (growth medium of inoculation), name of the lichen, name of the associated lichenicolous fungus, culture collection number and the newly published NCBI accession numbers are reported (bold). The affiliation (clade name) of the isolates is reported. Dash (-) indicates loss of culture due to unsuccessful subsequent growth. (DOCX 19 kb)

Table S4. List of Leotiomycetes and Sordariomycetes taxa retrieved from GenBank and selected for the phylogenetic analysis of Fig. S1 and Fig. S2. ID (if available) and NCBI accession numbers are reported. Outgroups are labelled by an asterisk. (DOCX 21 kb)

Table S5. Information about sizes of the genetic datasets before and after removing introns and ambiguous SNPs. The mitochondrial locus 16S was not used in the datasets of Leotiomycetes and Sordariomycetes. The original datasets of the nuclear 28S and 18S of Dothideomycetes were retrieved from previously composed datasets (Muggia et al. 2013) already trimmed from introns. (DOCX 13 kb)
