# Supplementary figures and images for: Extremotolerant fungi from alpine rock lichens and their phylogenetic relationships
Source: Fungal Divers. 2015 Aug 22;76:119–42. doi: 10.1007/s13225-015-0343-8 (PMC4739527; doi:10.1007/s13225-015-0343-8)

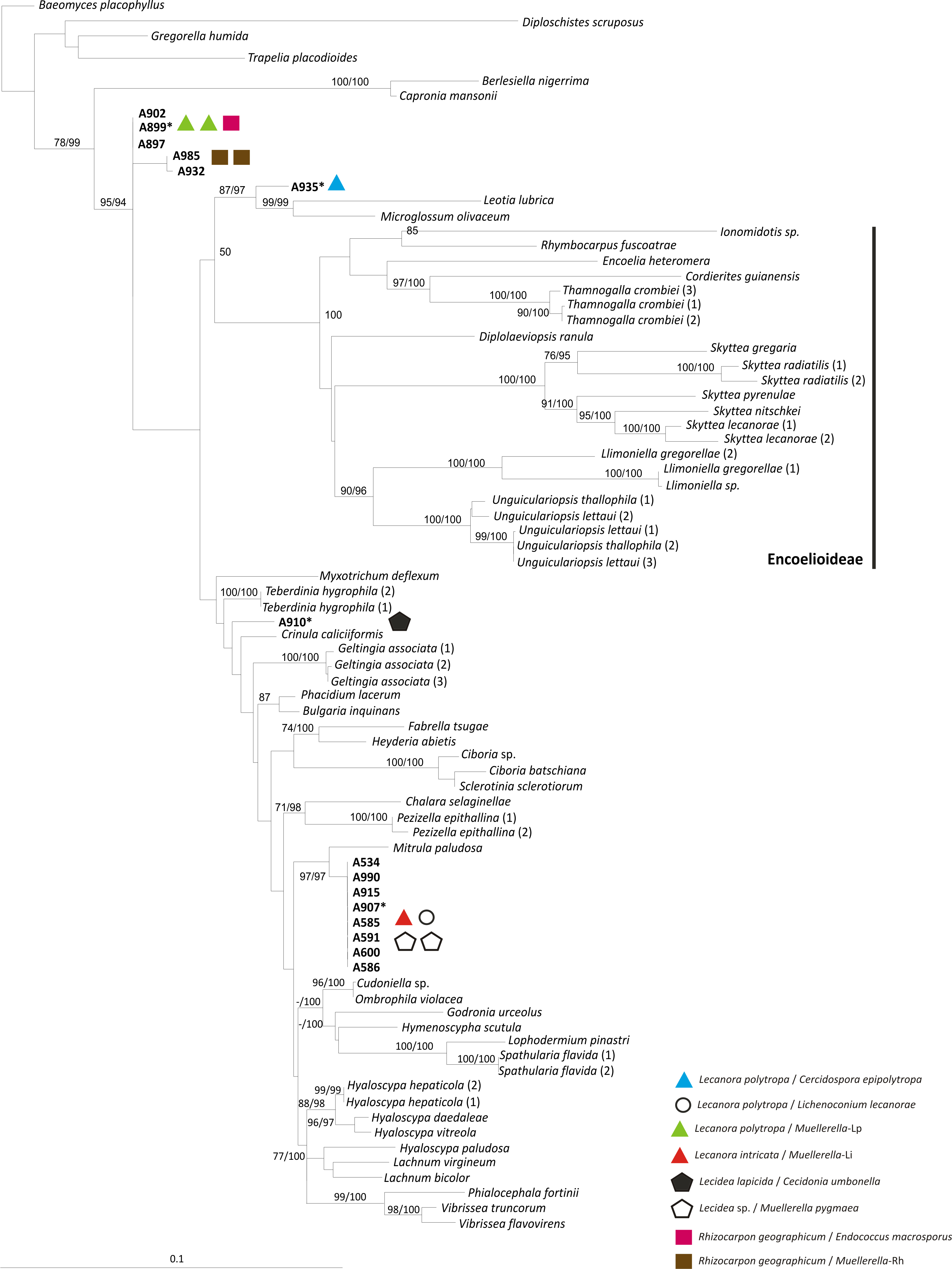

Supplement: Supplementary file 1 — Multilocus phylogenetic inference of Leotiomycetes. The ML and the Bayesian phylogenetic hypotheses were inferred from the combined dataset of nucLSU and nucSSU loci and corresponded in their topologies; the ML analysis is shown. ML bootstrap support values (≥ 70%) and Bayesian posterior probabilities (PP ≥ 95%) are reported above branches (bootstrap value/PP). Fungal isolates obtained from this study are highlighted in bold. Symbols indicate the different lichen host-lichenicolous fungal associations as reported in the legend. Samples labelled with an asterisk (*) are those photographed in Fig. S3. (JPEG 2367 kb) [file 13225_2015_343_Fig5_ESM.jpg]

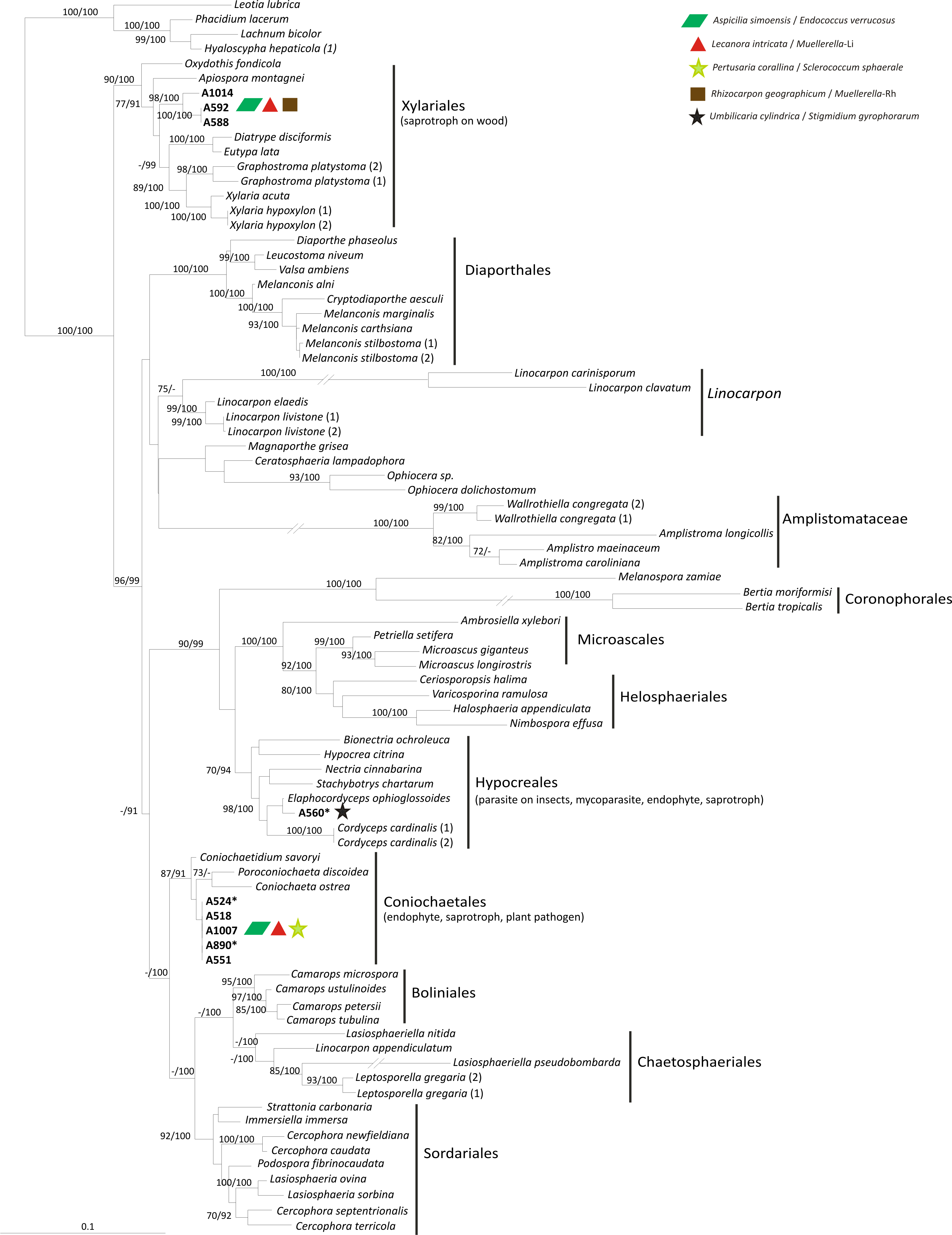

Supplement: Supplementary file 2 — Multilocus phylogenetic inference of Sordariomycetes. The ML and the Bayesian phylogenetic hypotheses were inferred from the combined dataset of nucLSU and nucSSU loci and corresponded in their topologies; the ML analysis is shown. ML bootstrap support values (> 70%) and Bayesian posterior probabilities (PP > 95%) are reported above branches (bootstrap value/PP). Fungal isolates obtained from this study are highlighted in bold. Symbols indicate the different lichen host-lichenicolous fungal associations as reported in the legend. Fungal life-styles are reported in parenthesis. Samples labelled with an asterisk (*) are those photographed in Fig. S3. (JPEG 2947 kb) [file 13225_2015_343_Fig6_ESM.jpg]

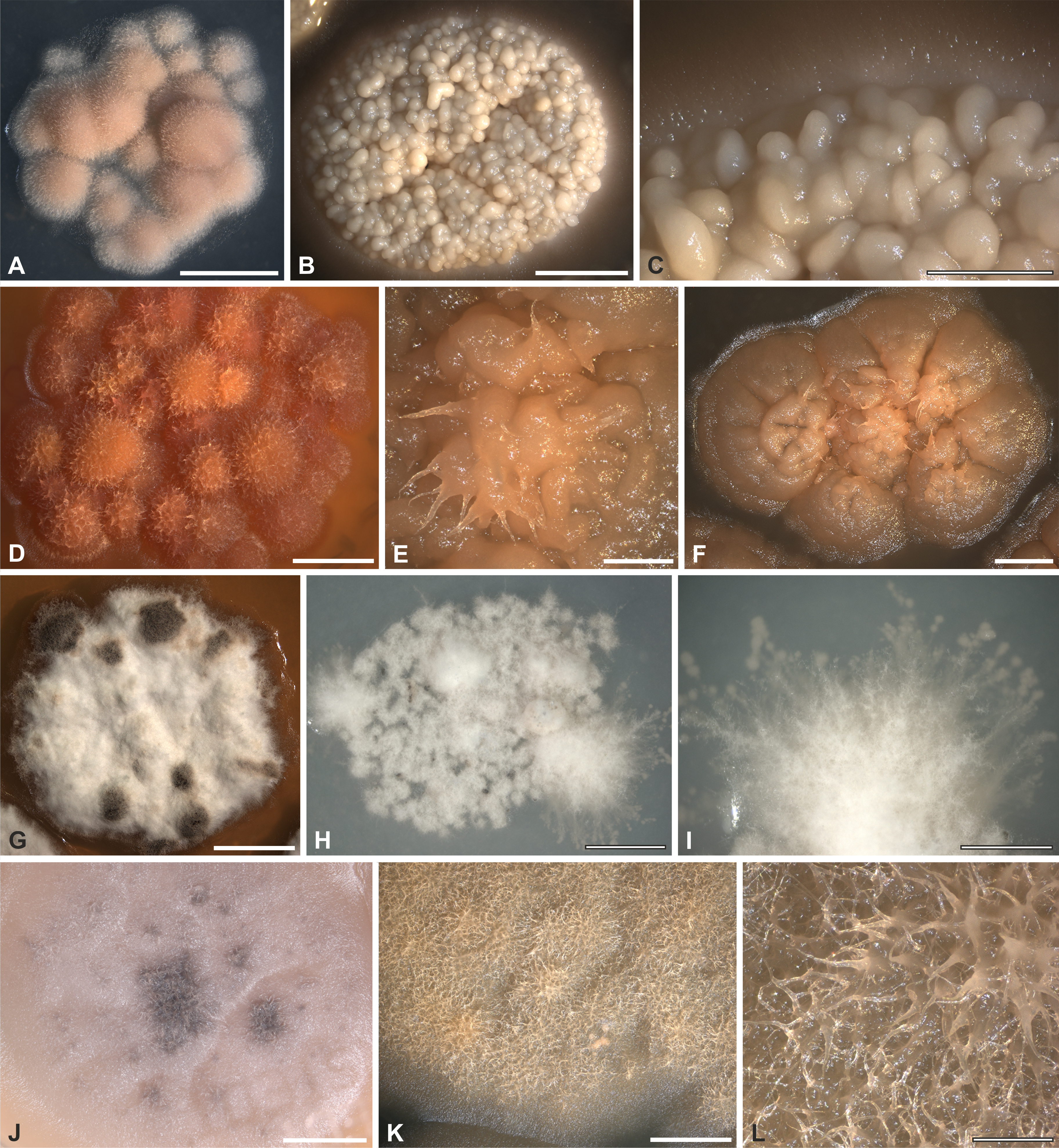

Supplement: Supplementary file 3 — Habitus of one year old, representative, cultured fungal strains belonging to Leotiomycetes and Sordariomycetes and included in the phylogenetic analysis of Fig. S1 and Fig. S2 respectively. Leotiomycetes: A) A899, B, C) A907, D) A910, E, F) A935. Sordariomycetes: G) A592 (Xylariales), H, I) A560 (Hypocreales), J) A524 (Coniochaetales), K, L) A890 (Coniochaetales). Sclae bars = 4 mm (A, B, D, F, G, H, J, K), 2 mm (C, I), 1 mm (E), 0.5 mm (L). (JPEG 8509 kb) [file 13225_2015_343_Fig7_ESM.jpg]
