# Supplementary material for: Extremotolerant fungi from alpine rock lichens and their phylogenetic relationships
Source: Fungal Divers. 2015 Aug 22;76:119–42. doi: 10.1007/s13225-015-0343-8 (PMC4739527; doi:10.1007/s13225-015-0343-8)
Supplement: Supplementary file 4 — List of Eurotiomycetes taxa retrieved from GenBank and selected for the phylogenetic analysis of Fig. 1. ID (if available) and NCBI accession numbers are reported. Outgroups are labelled by an asterisk. (DOCX 17 kb) [file 13225_2015_343_MOESM1_ESM.docx]

**Table S1.** List of Eurotiomycetes taxa retrieved from GenBank and selected for the phylogenetic analysis of Fig. 1. ID (if available) and NCBI accession numbers are reported. Outgroups are labelled by an asterisk.

|  |  |  |  |  |
| --- | --- | --- | --- | --- |
| **Taxon** | **Sample ID** | **nucLSU** | **nucSSU** | **mtSSU** |
|  |  |  |  |  |
|  |  |  |  |  |
| *Agonimia allobata* | L467 | FJ455771 | - | GU121589 |
| *Agonimia tristicula* | L469 (Hafellner 66664) | FJ455772 | - | GU12159 |
| *Anthracothecium nanum* | AFTOL 1649 | FJ358271 | FJ358339 | FJ225773 |
| *Arachniotus littoralis* | CBS 454.73 | FJ358272 | FJ358340 | FJ225773 |
| *Arachnomyces glareosum* | CBS 116.129 | FJ358273 | FJ358341 | FJ225785 |
| *Caliciopsis orientalis* | AFTOL 1911 | DQ470987 | DQ471039 | FJ190654 |
| *Caliciopsis pinea* | AFTOL 1869 | DQ678097 | DQ678043 | FJ190653 |
| *Capronia munkii* | AFTOL 656 | EF413604 | EF413603 | FJ225723 |
| *Capronia parasitica* | CBS 123.88 | FJ358225 | FJ358293 | FJ225724 |
| *Capronia peltigerae* |  | HQ613813 | HQ613815 | HQ613814 |
| *Capronia pillosella* | AFTOL 657 | DQ823099 | DQ823106 | FJ225725 |
| *Capronia semiimmersa* | AFTOL 658 | FJ358226 | - | FJ225726 |
| *Celothelium aciculiferum* | F16591 | DQ329019 | - | DQ328992 |
| *Celothelium cinchonarum* | F17105f | DQ329020 | - | DQ328993 |
| *Ceramothyrium carniolicum* (1) | CBS 175.95 | FJ358232 | FJ358300 | - |
| *Ceramothyrium carniolicum* (2) | AFTOL 1063 | EF413628 | EF413627 | - |
| *Chaenotheca savonica** |  | AY796000 | U86691 | - |
| *Cladophialophora arxii* |  | AB100683 | AJ232948 | - |
| *Cladophialophora devriesii* |  | AJ972912 | AJ232947 | - |
| *Cladophialophora minourae* | CBS 556.83 | FJ358235 | FJ358303 | - |
| *Cladophialophora parmeliae* (1) | Ertz 16591 | JX081671 | - | JX081675 |
| *Cladophialophora parmeliae* (2) | CBS 129.337 | JQ342182 | - | JQ342181 |
| *Endocarpon pallidum* | AFTOL 661 | DQ823097 | DQ823104 | FJ225674 |
| *Epibryon bryophilum* | M2 | EU940090 | EU940017 | EU940242 |
| *Epibryon hepaticola* | M10 | EU940091 | EU940018 | EU940243 |
| *Exophiala castellani* | CBS15858 | FJ358241 | JN856014 | FJ225739 |
| *Exophiala dermatitidis* | AFTOL 668 | DQ823100 | DQ823107 | - |
| *Exophiala oligosperma* | CBS 725.88 | FJ358245 | FJ358313 | FJ225743 |
| *Granulopyrenis seawardii* |  | EF411062 | EF411059 | - |
| *Heteroplacidium imbricatum* | AFTOL 2281 | EF643756 | EF689839 | FJ225679 |
| *Hydropunctaria maura* | AFTOL 2263 | EF643801 | - | FJ225681 |
| *Lithothelium septemseptatum* | AFTOL 12 | AY584638 | AY584662 | AY584620 |
| *Norrlinia peltigericola* |  | AY300845 | AY779280 | AY300896 |
| *Phialophora europaea* | CBS 129.96 | FJ358248 | FJ358317 | FJ225750 |
| *Placocarpus schaereri* | AFTOL 2289 | EF643766 | EF689850 | - |
| *Placopyrenium bucekii* | AFTOL 2238 | EF643768 | EF689852 | FJ225693 |
| *Pyrenula aspistea* (1) | GW1044 | JQ927470 | - | JQ927462 |
| *Pyrenula aspistea* (2) | AFTOL 2012 | EF411063 | EF411060 | - |
| *Pyrenula cruenta* |  | AF279407 | AF279406 | AY584719 |
| *Pyrenula macrospora* | CG1520a | JQ927473 | - | JQ927466 |
| *Pyrenula pseudobufonia* |  | AY640962 | AY641001 | AY584720 |
| *Pyrgillus javanicus* | AFTOL 342 | DQ823103 | DQ823110 | FJ225774 |
| *Schanorella spirotricha* | CBS 304.56 | FJ358288 | FJ358353 | FJ225793 |
| *Sclerococcum sphaerale* (1) | Diederich 17283 | JX081673 | - | - |
| *Sclerococcum sphaerale* (2) | Diederich 17279 | JX081672 | - | - |
| *Sclerococcum sphaerale* (3) | Ertz 17425 | JX081674 | - | - |
| *Sphinctrina turbinata** |  | EF413632 | EF413631 | FJ713611 |
| *Staurothele areolata* | AFTOL 2291 | EF643772 | EF689856 | FJ225699 |
| *Stenocybe pullatula** |  | AY796008 | SPU86692 | - |
| *Thelidium papulare* | AFTOL 2249 | EF643781 | EF689861 | DQ329005 |
| *Parabagliettoa dufourii* | AFTOL 2254 | EF643792 | EF689868 | FJ225684 |
| *Verrucaria viridula* | AFTOL 2299 | EF643814 | EF689884 | FJ22571 |
| *Verrucula inconnextaria* | AFTOL 307 | EF643821 | EF689892 | FJ225718 |
| rock isolate TRN1 |  | FJ358250 | FJ358319 | FJ225754 |
| rock isolate TRN14 |  | - | FJ358321 | FJ225756 |
| rock isolate TRN30 |  | FJ358252 | FJ358322 | FJ225757 |
| rock isolate TRN107 |  | FJ358253 | FJ358323 | FJ225758 |
| rock isolate TRN115 |  | FJ358254 | - | FJ225759 |
| rock isolate TRN210 |  | FJ358255 | FJ358325 | FJ225760 |
| rock isolate TRN214 |  | FJ358256 | - | FJ225761 |
| rock isolate TRN475 |  | FJ358260 | FJ358329 | FJ225764 |
| rock isolate TRN488 |  | FJ358262 | - | FJ225766 |
| rock isolate TRN493 |  | FJ358263 | FJ358331 | FJ225767 |
| rock isolate TRN497 |  | - | FJ358332 | FJ225768 |
| rock isolate TRN508 |  | FJ358265 | FJ358333 | FJ225770 |
| rock isolate TRN531 |  | FJ358267 | FJ358335 | FJ225772 |
|  |  |  |  |  |
