# Supplementary material for: Extremotolerant fungi from alpine rock lichens and their phylogenetic relationships
Source: Fungal Divers. 2015 Aug 22;76:119–42. doi: 10.1007/s13225-015-0343-8 (PMC4739527; doi:10.1007/s13225-015-0343-8)
Supplement: Supplementary file 5 — List of Dothideomycetes taxa retrieved from GenBank and selected for the phylogenetic analysis of Fig. 2. ID (if available) and NCBI accession numbers are reported. Outgroups are labelled by an asterisk. (DOCX 28 kb) [file 13225_2015_343_MOESM2_ESM.docx]

**Table S2.** List of Dothideomycetes taxa retrieved from GenBank and selected for the phylogenetic analysis of Fig. 2. ID (if available) and NCBI accession numbers are reported. Outgroups are labelled by an asterisk.

|  |  |  |  |  |
| --- | --- | --- | --- | --- |
| **Taxon** | **Sample ID** | **nucLSU** | **nucSSU** | **mtSSU** |
|  |  |  |  |  |
| *Abrothallus acetabuli* | SPO308 | KF816232 | KF816215 | KF816205 |
| *Abrothallus parmeliarum* | AB57 | KF816227 | KF816221 | - |
| *Abrothallus parmotrematis* | AB1 | KF816231 | KF816225 | - |
| *Abrothallus secedens* | SPO305 | KF816236 | KF816216 | KF816206 |
| *Abrothallus* sp. 2 | SP-2014 | KF816230 | KF816224 | KF816213 |
| *Acrospermium adeanum* | M133 | EU940104 | EU940031 | EU940256 |
| *Acrospermium compressum* | M151 | EU940084 | EU940012 | EU940237 |
| *Acrospermium gramineum* | M152 | EU940085 | EU940013 | EU940238 |
| *Anisomeridium phaeospermum* | MPN539 | JN887394 | JN887374 | - |
| *Anisomeridium polypori* | AFTOL 101 | - | DQ782877 | - |
| *Anisomeridium* sp. | MPN534 | JN887396 | JN887376 | JN887408 |
| *Anisomeridium* sp. | MPN542 | JN887398 | JN887378 | JN887410 |
| *Anisomeridium ubianum* |  | GU327709 | JN887379 | GU327682 |
| *Arthonia caesia** | AFTOL 775 | FJ469668 | - | FJ469671 |
| *Arthopyrenia salicis* | CBS 368.94 | AY538339 | AY538333 | AY779288 |
| *Asterina fuchsiae* | TH590 | GU586216 | GU586210 | - |
| *Asterina phenacis* | TH589 | GU586217 | GU586211 | - |
| *Bimuria novae-zelandae* | CBS 107.79/ AFTOL 931 | AY016356 | AY016338 | FJ190605 |
| *Botryosphaeria dothidea* | CBS 115.476/ AFTOL 946 | DQ678051 | DQ677998 | FJ190612 |
| *Botryosphaeria ribis* | AFTOL-ID 1232 | DQ678053 | DQ678000 | - |
| *Capnobotryella renispora* | CBS 214.90 | EU019248 | Y18698 | - |
| *Capnodium coffeae* | CBS 147.52/ AFTOL 939 | DQ247808 | DQ247801 | FJ190609 |
| Capnodiales sp. (Teratosphaeriaceae II) | L1285 | - | KC015090 | KR045763 |
| Capnodiales sp. (Teratosphaeriaceae II) | L1286 | - | KC015091 | KR045764 |
| *Catenulostoma abietis* | CBS 459.93/ AFTOL 2210 | DQ678092 | DQ678040 | - |
| *Cladosporium cladosporioides* | CBS 170.54/ AFTOL 1289 | DQ678057 | DQ678004 | FJ190628 |
| *Cladosporium* sp. | CBS 180.53/ AFTOL 1035 | AY016367 | AY016351 | AY350576 |
| *Coniosporium apollinis* | CBS 352.97 | GU250895 | GU250916 | GU250906 |
| *" "* | CBS 100.213 | GU250896 | GU250917 | GU250907 |
| *" "* | CBS 100.214 | GU250897 | GU250918 | GU250908 |
| *" "* | CBS 100.218 | GU250898 | GU250919 | GU250909 |
| *" "* | CBS 109.860 | GU250899 | GU250920 | GU250910 |
| *" "* | CBS 109.867 | GU250901 | - | GU250912 |
| *Coniosporium uncinatum* | CBS 100.219 | GU250903 | GU250923 | GU250914 |
| *" "* | CBS 100.212 | GU250902 | GU250922 | GU250913 |
| *Cryomyces antarcticus* | CCFEE 536 | GU250365 | GU250321 | GU250411 |
| *Cryomyces antarcticus* | CCFEE 456 | - | GU250316 | GU250405 |
| *Cryomyces minteri* | CBS 116302/ CCFEE 5187 | GU250369 | DQ066714 | GU250417 |
| *Cryomyces* sp. | CCFEE 5476 | GU250394 | GU250352 | GU250434 |
| *Cystocoleus ebeneus* | L348 (GZU, Hafellner 41566) | EU048580 | EU048573 | - |
| *Davidiella tassiana* | CBS 399.80/AFTOL 1591 (CPC11600 for mtSSU) | DQ678074 | DQ678022 | EU514455 |
| *Delphiniella strobiligena* | CBS 735.71/ AFTOL 1257 | DQ470977 | DQ471029 | - |
| *Dendrographa leucophaea f. minor** | AFTOL 355 | AF279382 | AF279381 | GU561843 |
| *Dendrographa leucophaea** | AFTOL 308 | AY548810 | AY548803 | AY571385 |
| *Dendryphiella arenaria* | CBS 181.58/ AFTOL 995 | DQ470971 | DQ471022 | FJ190617 |
| *Devriesia streliziae* | CBS 122379 | GU296146 | GU301810 | GU561845 |
| *Discosphaerina fagi* |  | AY016359 | - | AY016342 |
| *Dothidea insculpta* | CBS 189.58/ AFTOL 921 | DQ247802 | DQ247810 | FJ190602 |
| *Dothiora cannabinae* | CBS 737.71/ AFTOL 1359 | DQ470984 | DQ479933 | FJ190636 |
| *Elasticomyces elasticus* | CCFEE 5319 | GU250375 | GU250332 | - |
| *Elasticomyces elasticus* | CBS 122540/ CCFEE 5320 | GU250376 | GU250333 | GU250420 |
| *Elsinoe centrolobi* | CBS 222.50/ AFTOL 1854 | DQ678094 | DQ678041 | FJ190651 |
| *Elsinoe phaseoli* | CBS 165.31/ AFTOL 1855 | DQ678095 | DQ678042 | - |
| *Elsinoe veneta* | AFTOL1360 | DQ678060 | DQ678007 | - |
| *Elsinoe veneta* | AFTOL1853 | DQ767658 | DQ767651 | FJ190650 |
| *Etayoa trypethelii* | Common 9200G | KF176940 | - | KF176967 |
| *Etayoa trypethelii* | Common 9215P | KF176941 | - | KF176968 |
| *Etayoa trypethelii* | Mukherjee | KF176943 | - | KF176970 |
| *Flavobathelium epiphyllum* |  | GU327717 | JN887382 | - |
| *Friedmanniomyces endolithicus* | CCFEE 524 | GU250364 | DQ066715 | GU250409 |
| *Gibbera conferta* | CBS 191.53 | GU301814 | GU296150 | - |
| *Gloniopsis praelonga* | CBS 112.415 | FJ161173 | FJ161134 | - |
| *Guignardia bidwellii* | CBS 237.48/ AFTOL 1618 | DQ678085 | DQ678034 | AF271135 |
| *Helicomyces roseus* | CBS 283.51/ AFTOL 1613 | DQ678083 | DQ678032 | - |
| *Hysteropatella clavispora* | CBS 247.34/ AFTOL 1305 | AY541493 | DQ678006 | AY571388 |
| *Hysteropatella elliptica* | CBS 935.97/ AFTOL 1790 | DQ767657 | EF495114 | FJ190649 |
| *Kirschsteiniothelia aethiops* | CBS 109.53/ AFTOL 925 | AY016361 | AY016344 | FJ190604 |
| *Laurera megasperma* | AFTOL 2094 | FJ267702 | GU561841 | GU561847 |
| *Lecanactis abietina** | AFTOL 305 | AY548812 | AY548805 | AY548813 |
| *Leptosphaeria maculans* | DAOM 229267/ AFTOL 277 | DQ4709646 | DQ470993 | - |
| *Lichenoconium aeruginosum* | JL359-09 | HQ174269 | - | HQ174268 |
| *Lichenoconium erodens* | JL363-09 | HQ174267 | - | HQ174266 |
| *Lichenoconium lecanorae* | JL382-10 | HQ174263 | - | HQ174262 |
| *Lichenoconium usneae* | JL352-09 | HQ174265 | - | HQ174264 |
| *Lichenostigma alpinum* | Ertz 17522 | KF176945 | - | KF176972 |
| *Lichenostigma alpinum* | Ertz 17519 | KF176946 | - | KF176973 |
| *Lichenostigma chlaroterae* | Diederich 17329 | KF176947 | - | KF176974 |
| *Lichenostigma chlaroterae* | Neuberg | KF176948 | - | KF176975 |
| *Lichenostigma maureri* | Diederich 17306 | KF176951 | - | KF176978 |
| *Lichenostigma maureri* | Diederich 17337 | KF176952 | - | KF176980 |
| *Lichenostigma* sp. | Diederich 17240 | KF176955 | - | KF176981 |
| Dothideomycetes incerta saedis | L1854 | KR045757 | KR045791 | KR045767 |
| Dothideomycetes incerta saedis | L1855 | KR045758 | KR045767 | KR045768 |
| *Lichenothelia arida* | L2024 | - | KR045796 | KR045771 |
| *Lichenothelia arida* | L1703 | - | KR045799 | KR045772 |
| *Lichenothelia calcarea* | L1799 | KR045746 | KR045804 | KR045775 |
| *Lichenothelia convexa* | Diederich 17491 | KF176962 | - | KF176988 |
| *Lichenothelia convexa* | L1607 | KC015069 | KC015084 | - |
| *Lichenothelia convexa* | L1608 | KC015070 | KC015085 | - |
| *Lichenothelia convexa* | L1609 | KC015071 | KC015086 | - |
| *Lichenothelia rugosa* | Ertz 16065 | KF176964 | - | - |
| *Lichenothelia rugosa* | Diederich 17310 | KF176963 | - | KF176989 |
| *Lichenothelia* sp. | Ertz 15255 | KF176950 | - |  |
| *Lichenothelia* sp. | Ertz 16122 | KF176956 | - | KF176982 |
| *Lichenothelia* sp. | Ertz 16340 | KF176957 | - | KF176983 |
| *Lichenothelia* sp. | Ertz 16455 | KF176958 | - | KF176984 |
| *Lichenothelia* sp. | L984 | KC015074 | KC015087 | - |
| *Lichenothelia* sp. | L985 | KC015075 | KC015088 | - |
| *Lichenothelia* sp. | L986 | KC015076 | KC015089 | KR045782 |
| *Lichenothelia tenuissima* | L1798 (Knudsen K. 10406) | KC015073 | **-** | - |
| *Lichenothelia umbrophila* | L1323 | KC015061 | KC015081 | - |
| *Lichenothelia umbrophila* | L1324 | KC015062 | KC015082 | - |
| *Lophium mytilinum* | CBS 269.34/ AFTOL 1609 | DQ678081 | DQ678030 | - |
| *Macrophomina phaseolina* | CBS 227.33/ AFTOL 1783 | DQ678088 | DQ678037 | - |
| *Megalotremis verrucosa* | Lucking 26316 (F) | GU327718 | JN887383 | - |
| *Microxyphium citri* | CBS 451.66 | GU301848 | GU296177 | AF346421 |
| *Mycosphaerella euripotami* | JK 5586J | GU301852 | GZ479761 | GU566746 |
| *Mycosphaerella fijiensis* | OSC 100622/ AFTOL 2021 | DQ678098 | DQ767652 | FJ190656 |
| *Myriangium duriaei* | CBS 260.36/ AFTOL 1304 | DQ678059 | AY016347 | AY350575 |
| *Mytilinidion resinicola* | CBS 304.34 | FJ161185 | FJ161145 | - |
| *Patellaria atrata* | CBS 958.97 | GU301855 | GU296181 | - |
| *Phaeosclera dematioides* | CBS 157.81 | GU301858 | GU296184 | - |
| *Phaeotrichum benjaminii* | CBS 541.72/ AFTOL 1184 | AY004340 | AY016348 | AY538349 |
| *Phoma caloplacae* | CBS 129.338 | JQ238643 | - | JQ238642 |
| *Phoma caloplacae* | CBS 129.140 | JQ238637 | - | JQ238636 |
| *Phoma cladoniicola* (1) | CBS 128.026 | JQ238628 | - | JQ238627 |
| *Phoma cladoniicola* (2) | CBS 128.023 | JQ238622 | - | JQ238621 |
| *Phoma cladoniicola* (3) | FL14 | JQ318028 | - | JQ318027 |
| *Phyllobathelium anomalum* |  | GU327722 | JN887386 | GU327698 |
| *Phyllobathelium firmum* | MPN545 | JN887401 | JN887387 | JN887413 |
| *Pleospora herbarum* | CBS 541.72/ AFTOL 940 | DQ247804 | DQ247812 | FJ190610 |
| *Preussia terricola* | DAOM 230091/ AFTOL 282 | AY544686 | AY544726 | AY544754 |
| *Rachicladosporium mcmurdoii* | CCFEE 5211 | GU250371 | GU250419 | GU250419 |
| *Racodium rupestre* | L424 (TSB 37932) | EU048582 | EU048577 | EU048589 |
| *Ramularia graminicola* | CBS 292.38/ AFTOL 1615 | DQ678084 | DQ678033 | - |
| *Ramularia punctiformis* | CBS 113265/ AFTOL 942 | DQ470968 | DQ471017 | FJ190611 |
| *Recurvomyces mirabilis* | CCFEE 5475 | KC315876 | KC315865 | KC315887 |
| *Recurvomyces mirabilis* | CCFEE 5264 | GU250372 | GU250329 | - |
| *Rhytidisterium rufulum* | CBS 306.38 | FJ469672 | AF164375 | - |
| *Roccella fuciformis** | AFTOL 126 (Diederich 15572 for mtSSU) | AY584654 | AY584678 | EU704082 |
| *Roccellographa cretacea** | AFTOL 93 | DQ883696 | DQ883705 | FJ772240 |
| *Saxomyces alpinus* | CCFEE 5469 | KC315871 | KC315860 | KC315882 |
| *Saxomyces alpinus* | CCFEE 5470 | KC315872 | KC315861 | KC315883 |
| *Saxomyces penninicus* | CCFEE 5495 | KC315875 | KC315864 | KC315886 |
| *Schismatomma decolorans** | AFTOL 307 | AY548815 | AY548809 | AY548816 |
| *Scorias spongiosa* | CBS 325.33/ AFTOL 1594 | DQ678075 | DQ678024 | FJ190643 |
| *Simonyella variegata** | AFTOL 80 | - | AY584669 | AY584631 |
| *Sirodesmium olivaceum* | CBS 395.59 | GU250915 | GU250904 | GU250904 |
| *Strigula jamesii* | MPN548 | JN887404 | JN887388 | JN887416 |
| *Strigula nemathora* | MPN72 | JN887405 | JN887389 | GU327701 |
| *Stylodothis puccinioides* | CBS 193.58 | AY004342 | AY016353 | AF346428 |
| *Sydowia polyspora* | CBS 116.29/ AFTOL 1300 | DQ678058 | DQ678005 | FJ190631 |
| *Teratosphaeria associata* | CBS 112224 | GU301874 | GU296200 | - |
| *Tripospermum myrti* | CBS 437.68 | GU323216 | - | - |
| *Trypethelium nitidiusculum* | AFTOL 2099 | FJ267701 | GU561842 | GU561848 |
| *Trypethelium* sp. | AFTOL 110 | AY584652 | AY584676 | AY584632 |
| *Tubeufia cerea* | CBS 254.75/ AFTOL 1316 | DQ470982 | DQ471034 | FJ190634 |
| *Tubeufia paludosa* | CBS 245.49/ AFTOL 1589 | DQ767654 | DQ767649 | GU566745 |
| *Tyrannosorus pinicola* | CBS 124.88/ AFTOL 1235 | DQ470974 | DQ471025 | FJ190620 |
| *Venturia inaequalis* | CBS 594-70 | GU301879 | NG016539 | - |
| *Venturia inaequalis* | ATCC60070 | EF114712 | EF114737 | - |
| *Westerdykella cylindrica* | CBS 454.72/ AFTOL 1037 | AY004343 | AY016355 | AF346430 |
| rock isolate TRN5 (Dothideomycetes sp.) | CBS 118.762 | GU323956 | GU323988 | GU324017 |
| rock isolate TRN11 (Dothideomycetes sp.) | CBS 118.281 | GU323957 | - | GU324018 |
| rock isolate TRN42 (Dothideomycetes sp.) | CBS 117958 | GU323958 | - | GU324019 |
| rock isolate TRN62 (Dothideomycetes sp.) | CBS 118.305 | GU323961 | GU323991 | GU324022 |
| rock isolate TRN66 (Dothideomycetes sp.) | CBS 118.306 | GU323962 | GU323992 | GU324023 |
| rock isolate TRN77 (Dothideomycetes sp.) | CBS 118.287 | GU323963 | GU323993 | GU324024 |
| rock isolate TRN87 (Dothideomycetes sp.) | CBS 118.290 | GU323966 | GU323996 | GU324027 |
| rock isolate TRN111 (Dothideomycetes sp.) | CBS 118.294 | GU323967 | GU324028 | GU324028 |
| rock isolate TRN123 (Dothideomycetes sp.) | CBS 117.932 | GU323970 | GU323999 | GU324031 |
| rock isolate TRN124 (Dothideomycetes sp.) | CBS 118.283 | GU323971 | GU324000 | GU324032 |
| rock isolate TRN137 (Dothideomycetes sp.) | CBS 118.300 | GU323973 | GU324002 | GU324034 |
| rock isolate TRN138 (Dothideomycetes sp.) | CBS 118.301 | GU323974 | GU324003 | GU324035 |
| rock isolate TRN142 (Dothideomycetes sp.) | CBS 118.302 | GU323975 | GU324004 | GU324036 |
| rock isolate TRN153 (Dothideomycetes sp.) | CBS 118.330 | GU323977 | GU324006 | GU324038 |
| rock isolate TRN235 (Dothideomycetes sp.) | CBS 118.605 | GU323979 | - | GU324041 |
| rock isolate TRN267 (Dothideomycetes sp.) | CBS 118.769 | - | GU324043 | GU324043 |
| rock isolate TRN268 (Dothideomycetes sp.) | CBS 119.305 | GU323981 | - | GU324044 |
| rock isolate TRN456 (Dothideomycetes sp.) | - | GU323986 | GU324015 | GU324049 |
| rock isolate TRN529 (Dothideomycetes sp.) | - | GU323987 | GU324016 | GU32405 |
|  |  |  |  |  |
