# Supplementary material for: Extremotolerant fungi from alpine rock lichens and their phylogenetic relationships
Source: Fungal Divers. 2015 Aug 22;76:119–42. doi: 10.1007/s13225-015-0343-8 (PMC4739527; doi:10.1007/s13225-015-0343-8)
Supplement: Supplementary file 6 — List of Leotiomycetes and Sordariomycetes isolates obtained in this study and included in the analyses of Fig. S1 and Fig. S2 respectively. Number of the original lichen thallus (growth medium of inoculation), name of the lichen, name of the associated lichenicolous fungus, culture collection number and the newly published NCBI accession numbers are reported (bold). The affiliation (clade name) of the isolates is reported. Dash (-) indicates loss of culture due to unsuccessful subsequent growth. (DOCX 19 kb) [file 13225_2015_343_MOESM3_ESM.docx]

**Table S3.** List of Leotiomycetes and Sordariomycetes isolates obtained in this study and included in the analyses of Fig. S1 and Fig. S2 respectively. Number of the original lichen thallus (growth medium of inoculation), name of the lichen, name of the associated lichenicolous fungus, culture collection number and the newly published NCBI accession numbers are reported. The affiliation (clade name) of the isolates is reported. Dash (-) indicate loss of culture due to unsuccessful subsequent growth.

|  |  |  |  |  |  |  |  |  |
| --- | --- | --- | --- | --- | --- | --- | --- | --- |
| **lichen thallus ID (medium name)** | **lichen species** | **lichenicolous fungus species** | **cultured fungus DNA extraction N.** | **culture collection N.** | **nucLSU** | **nucSSU** | **mtSSU** | **phlogenetic clade** |
|  |  |  |  |  |  |  |  |  |
|  |  |  |  |  |  |  |  |  |
| A65 (LBM) | *Lecanora polytropa* | *Muellerella* - Lp | A899 | LMCC0255 | **KT263282** | **KT263301** | **KT263272** | - |
| A94 (DG) | *Lecanora intricata* | *Muellerella* - Li | A585 | LMCC0194 | **KT263285** | **KT263296** | **KT263267** | - |
| A94 (DG) | *Lecanora intricata* | *Muellerella* - Li | A586 | - | **KT263286** | **KT263297** | **KT263268** | - |
| A94 (MY) | *Lecanora intricata* | *Muellerella* - Li | A591 | LMCC0198 | **KT263294** | **KT263299** | **KT263280** | - |
| A173 (KGA) | *Lecanora polytropa* | *Lichenoconium lecanorae* | A534 | LMCC0152 | **KT263284** | **KT263295** | **KT263269** | - |
| A193 (KGA) | *Lecidea lapicida* | *Muellerella pygmaea* | A907 | LMCC0227 | **KT263288** | **KT263303** | **KT263274** | - |
| A201 (TM) | *Lecidea* sp. | *Muellerella pygmaea* | A600 | LMCC0207 | **KT263287** | **KT263298** | **KT263270** | - |
| A201 (DG) | *Lecidea* sp. | *Muellerella pygmaea* | A915 | LMCC0258 | **KT263289** | **KT263305** | **KT263276** | - |
| A263 (TM) | *Rhizocarpon geographicum* | *Muellerella* - Rh | A932 | LMCC0273 | **-** | **KT263306** | **KT263281** | - |
| A268 (KGA) | *Rhizocarpon geographicum* | *Endococcus macrosporus* | A897 | LMCC0254 | **KT263293** | **KT263300** | **KT263271v** | - |
| A319 (MY) | *Lecidea* sp. | *Muellerella pygmaea* | A990 | LMCC0322 | **KT263290** | **KT263308** | **KT263278** | - |
| A337 (KGA) | *Lecanora polytropa* | *Cercidospora epipolytropa* | A935 | LMCC0306 | **KT263292** | **KT263307** | **KT263277** | - |
| A385 (SAB) | *Rhizocarpon geographicum* | *Muellerella* - Rh | A985 | LMCC0319 | **-** | **KT263309** | **KT263279** | - |
| A398 (SAB) | *Lecidea lapicida* | *Cecidonia umbonella* | A910 | LMCC0229 | **KT263291** | **KT263304** | **KT263275** | - |
| A426 (KGA) | *Lecanora polytropa* | *Muellerella* - Lp | A902 | LMCC0222 | **KT263283** | **KT263302** | **KT263273** | - |
|  |  |  |  |  |  |  |  |  |
|  |  |  |  |  |  |  |  |  |
| A65 (SAB) | *Lecanora intricata* | *Muellerella* - Li | A890 | LMCC0252 | **KT263314** | **KT263325** | **KT263262** | Coniochaetales |
| A77 (SAB) | *Aspicilia simoensis* | *Endococcus verrucosus* | A518 | LMCC0139 | **KT263315** | **KT263319** | **KT263259** | Coniochaetales |
| A77 (SAB) | *Aspicilia simoensis* | *Endococcus verrucosus* | A524 | LMCC0144 | **KT263316** | **KT263320** | **KT263260** | Coniochaetales |
| A77 (MY) | *Aspicilia simoensis* | *Endococcus verrucosus* | A551 | LMCC0231 | **KT263317** | **KT263321** | **KT263261** | Coniochaetales |
| A90 (SAB) | *Aspicilia simoensis* | *Endococcus verrucosus* | A592 | LMCC0199 | **KT263312** | **KT263324** | **KT263266** | Xylariales |
| A94 (TM) | *Lecanora intricata* | *Muellerella* - Li | A588 | LMCC0196 | **KT263313** | **KT263323** | **KT263265** | Xylariales |
| A100 (LBM) | *Umbilicaria cylindrica* | *Stigmidium gyrophorarum* | A560 | LMCC0171 | **KT263318** | **KT263322** | **KT263264** | Hypocreales |
| A263 (DG) | *Rhizocarpon geographicum* | *Muellerella* - Rh | A1014 | LMCC0339 | **KT263311** | **KT263327** | **-** | Xylariales |
| A774 (TM) | *Pertusaria corallina* | *Sclerococcum sphaerale* | A1007 | LMCC0336 | **KT263310** | **KT263326** | **KT263263** | Coniochaetales |
