# Supplementary material for: Extremotolerant fungi from alpine rock lichens and their phylogenetic relationships
Source: Fungal Divers. 2015 Aug 22;76:119–42. doi: 10.1007/s13225-015-0343-8 (PMC4739527; doi:10.1007/s13225-015-0343-8)
Supplement: Supplementary file 7 — List of Leotiomycetes and Sordariomycetes taxa retrieved from GenBank and selected for the phylogenetic analysis of Fig. S1 and Fig. S2. ID (if available) and NCBI accession numbers are reported. Outgroups are labelled by an asterisk. (DOCX 21 kb) [file 13225_2015_343_MOESM4_ESM.docx]

**Table S4.** List of Leotiomycetes and Sordariomycetes taxa retrieved from GenBank and selected for the phylogenetic analysis of Fig. S1 and Fig. S2. ID (if available) and NCBI accession numbers are reported. Outgroups are labelled by an asterisk.

|  |  |  |  |
| --- | --- | --- | --- |
| **Taxon** | **Sample ID** | **nucLSU** | **nucSSU** |
|  |  |  |  |
| **Leotiomycetes** |  |  |  |
| *Baeomyces placophyllus** |  | AF356658 | AF356657 |
| *Berlesiella nigerrima** | CBS51369 | AY350579 | AY541478 |
| *Bulgaria inquinans* | ZWGeo 52 Clark | AY789344 | AY789343 |
| *Capronia mansonii** | CBS10167 | AY004338 | X79318 |
| *Chalara selaginellae* | OC0011 | FJ176241 | FJ176266 |
| *Ciboria batschiana* |  | - | DQ257354 |
| *Ciboria* sp. | WZJXD22 | AY789322 | - |
| *Cordierites guianensis* | 192 | EU107270 | EU107262 |
| *Crinula caliciiformis* | AFTOL 27228S | AY544680 | AY544729 |
| *Cudoniella* sp. | ZW0068 | AY789341 | AY789340 |
| *Diplolaeviopsis ranula* | Diederich 16989 | KJ559554 | - |
| *Diploschistes scruposus** |  | AF279389 | AF279388 |
| *Encoelia heteromera* | 195 | EU107233 | EU107204 |
| *Fabrella tsugae* |  | AF356694 | AF106015 |
| *Geltingia associata* (1) | Perez-Ortega 1039 | KJ559562 | KJ559584 |
| *Geltingia associata* (2) | Bjoerk 22459 | KJ559576 | KJ559580 |
| *Geltingia associata* (3) | TU 45655 | KJ559564 | KJ559585 |
| *Godronia urceolus* | CBS 215.58 | - | EU754065 |
| *Gregorella humida** | Pykala 23630 | EU360846 | EU360867 |
| *Heyderia abietis* | OSC60392 | AY789289 | AY789288 |
| *Hyaloscypha daedaleae* | ZWGeo 138 Clark | AY789415 | AY789414 |
| *Hyaloscypha hepaticola* (1)^#^ | M171 | EU940118 | EU940045 |
| *Hyaloscypha hepaticola* (2) | M339 | EU940150 | EU940074 |
| *Hyaloscypha paludosa* | ATL2012/ M229 | EU940138 | EU940064 |
| *Hyaloscypha vitreola* | M39 | EU940155 | EU940079 |
| *Hymenoscypha scutula* | MBH29259 | AY789431 | AY789430 |
| *Ionomidotis* sp. | 190 | EU107271 | EU107263 |
| *Lachnum bicolor^#^* | OSC100069/ AFTOL 177 | AY544674 | AY544690 |
| *Lachnum virgineum* | AFTOL 49/ spat 0301 | AY544646 | AY544688 |
| *Leotia lubrica^#^* | ZW Geo59 Clark | AY789359 | AY789358 |
| *Llimoniella gregorellae* (1) | CBFS JV 9954 | KJ559553 | KJ559581 |
| *Llimoniella gregorellae* (2) | Vondrak 8374 | KJ559569 | KJ559589 |
| *Llimoniella* sp. | Diederich 17525 | KJ559555 | - |
| *Lophodermium pinastri* |  | AY004334 | - |
| *Microglossum olivaceum* | FHDSH97103_ | AY789397 | - |
| *Mitrula paludosa* | MBH50636 | AY789423 | - |
| *Myxotrichum deflexum* |  | AY541491 | - |
| *Ombrophila violacea* | WZ0024 | AY789365 | - |
| *Pezizella epithallina* (1) | Diederich 17411 | KJ559572 | - |
| *Pezizella epithallina* (2) | TU 39378 | KJ559570 | KJ559590 |
| *Phacidium lacerum^#^* | AFTOL 1253 | DQ470976 | DQ471028 |
| *Phialocephala fortinii* | CMW815 | AF269219 | AY524846 |
| *Rhymbocarpus fuscoatrae* | Ertz16200 | KJ559571 | KJ559593 |
| *Sclerotinia sclerotiorum* | WZ0067 | AY789347 | AY789346 |
| *Skyttea gregaria* | NY0118113 | KJ559559 | - |
| *Skyttea lecanorae* | NY1595972 | KJ559561 | - |
| *Skyttea nitschkei* | Ertz 17483 | KJ559577 | KJ559595 |
| *Skyttea radiatilis* (1) | NY00977030 | KJ559558 | - |
| *Skyttea radiatilis* (2) | NY0123276 | KJ559560 | - |
| *Skyttea lecanorae* | Ertz 16099 | KJ559574 | KJ559597 |
| *Skyttea pyrenulae* | Ertz 16253 | KJ559575 | KJ559596 |
| *Spathularia flavida* (1) | WZ138 | AF433142 | - |
| *Spathularia flavida* (2) |  | AY541496 | - |
| *Teberdinia hygrophila* (1) | 229 | JQ780654 | JQ780655 |
| *Teberdinia hygrophila* (2) | 150 | JQ780642 | JQ780643 |
| *Thamnogalla crombiei* (1) | Diederich 17553 | KJ559557 | KJ559583 |
| *Thamnogalla crombiei* (2) | Diederich 17544 | KJ559556 | - |
| *Thamnogalla crombiei* (3) | Diederich 17315 | KJ559578 | KJ559594 |
| *Trapelia placodioides** |  | AF274103 | AF119500 |
| *Unguiculariopsis lettaui* (1) | TU45124 | KJ559563 | - |
| *Unguiculariopsis lettaui* (2) | TU64867 | KJ559566 | KJ559586 |
| *Unguiculariopsis lettaui* (3) | Ertz 16346 | KJ559579 | KJ559592 |
| *Unguiculariopsis thallophila* (1) | TU45703 | KJ559565 | - |
| *Unguiculariopsis thallophila* (2) | Diederich 16944 | KJ559573 | - |
| *Vibrissea flavovirens* | MBH39316 | AY789426 | AY789425 |
| *Vibrissea truncorum* | CUP62562 | AY789402 | AY789401 |
|  |  |  |  |
| **Sordariomycetes** |  |  |  |
| *Ambrosiella xylebori* | AFTOL 1285 | DQ470979 | DQ471031 |
| *Apiospora montagnei* | AFTOL 951 | DQ471018 | JN546134 |
| *Amplistroma caroliniana* | DOI | FJ532376 | - |
| *Amplistroma einaceum* | AH43902 | KC907374 | - |
| *Amplistroma longicollis* | AH37870 | HQ901790 | - |
| *Bertia moriformis* | SMH4320 | AY695260 | - |
| *Bertia tropicalis* | SMH1707 | AY695262 | - |
| *Bionectria ochroleuca* | CCFC226708/ AFTOL 187 | AY283558 | DQ862044 |
| *Camarops microspora* |  | AY083821 | AY083800 |
| *Camarops petersii* |  | AY346265 |  |
| *Camarops tubulina* |  | AY346266 |  |
| *Camarops ustulinoides* | AFTOL 72 | DQ470941 | DQ470989 |
| *Ceratosphaeria lampadophora* |  | AY346270 | AY761088 |
| *Cercophora caudata* | CBS 606.72 | AY999113 | DQ368659 |
| *Cercophora newfieldiana* | SMH3303 | AY780062 | - |
| *Cercophora septentrionalis* |  | U47823 | U32400 |
| *Cercophora terricola* | ATCC 200395 | AY780067 |  |
| *Ceriosporopsis halima* |  | U47844 | U47843 |
| *Coniochaeta ostrea* | AFTOL915 | DQ470959 | DQ471007 |
| *Coniochaetidium savoryi* |  | AY346276 | - |
| *Cordyceps cardinalis* (1) | OSC93620 | AY184965 | AY184976 |
| *Cordyceps cardinalis* (2) | OSC93619 | AY184964 | AY184975 |
| *Cryptodiaporthe aesculi* | AFTOL 1238 | DQ836905 | DQ836899 |
| *Diaporthe phaseolorum* |  | AY346279 | - |
| *Diatrype disciformis* | AFTOL 927 | DQ470964 | DQ471012 |
| *Elaphocordyceps ophioglossoides* | CBS 100.239 | KJ878874 | KJ878910 |
| *Eutypa lata* | AFTOL 929 | DQ836903 | DQ836896 |
| *Graphostroma platystoma* (1) | AFTOL 1249 | DQ836906 | DQ836900 |
| *Graphostroma platystoma* (2) |  | AY083827 | AY083808 |
| *Halosphaeria appendiculata* |  | U46885 | U46872 |
| *Hypocrea citrina* |  | EU481408 | AY779279 |
| *Immersiella immersa* | SMH2589 | AY436408 | - |
| *Lasiosphaeria ovina* | SMH3923/ M176 | AY587950 | EU940082 |
| *Lasiosphaeria sorbina* | GJSL555 | AY436415 | - |
| *Lasiosphaeriella nitida* | SMH1290 | HM171283 | - |
| *Lasiosphaeriella pseudobombarda* | SMH4365 | HM171285 | - |
| *Leptosporella gregaria* (1) |  | AY346290 | - |
| *Leptosporella gregaria* (2) | SMH4867 | HM171288 | - |
| *Leucostoma niveum* | AR3512 | AF408367 | - |
| *Linocarpon appendiculatum* | HKUCC2986 | DQ810199 | - |
| *Linocarpon carinisporum* | HKUM7710 | DQ810200 | - |
| *Linocarpon clavatum* | HKUM1924 | DQ810201 | - |
| *Linocarpon elaeidis* | 5458 | DQ810222 | DQ810257 |
| *Linocarpon livistonae* (1) | HKUCC2954 | DQ810206 | - |
| *Linocarpon livistonae* (2) | HKUM6520 | DQ810205 | - |
| *Magnaporthe grisea* | AR3390 | AF362554 | - |
| *Melanconis alni* | AFTOL2127 | - | DQ862052 |
| *Melanconis carthusiana* | AR3581 | EU255135 | - |
| *Melanconis marginalis* |  | AF277144 | AF277122 |
| *Melanconis stilbostoma* (1) | AFTOL 936 | FJ713618 | DQ862043 |
| *Melanconis stilbostoma* (2) | AR3501 | AF408374 | - |
| *Melanconis stilbostoma* (3) | E01051 | AY577813 | AY577812 |
| *Melanospora zamiae* | ATCC 96173 | AY057906 | AY057905 |
| *Microascus giganteus* |  | AF275540 | - |
| *Microascus longirostris* | AFTOL1237 | - | DQ471026 |
| *Microascus trigonosporus* | AFTOL 914 | DQ470958 | DQ471006 |
| *Nectria cinnabarina* |  | AF193237 | U32412 |
| *Nimbospora effusa* |  | U46892 | U46877 |
| *Ophioceras* sp. | CMU 26633 | EU571272 | EU571271 |
| *Ophioceras dolichostomum* | CBS 114.926 | JX134689 | JX134663 |
| *Oxydothis fondicola* |  | AY083835 | AY083818 |
| *Petriella setifera* | AFTOL 956 | DQ470969 | DQ471020 |
| *Podospora fibrinocaudata* | TRTC 48343 | AY780074 | - |
| *Poroconiochaeta discoidea* |  | AY346297 | - |
| *Strattonia carbonaria* |  | AY346302 | - |
| *Stachybotrys chartarum* | CBS 363.49 | AY554249 | - |
| *Valsa ambiens* | AFTOL 2131 | AF277146 | DQ862056 |
| *Varicosporina ramulosa* |  | U44092 | U43846 |
| *Wallrothiella congregata* (1) | SMH1760 | FJ532375 | - |
| *Wallrothiella congregata* (2) | ANM81 | FJ532374 | - |
| *Xylaria acuta* | AFTOL 63 | AY544676 | AY544719 |
| *Xylaria hypoxylon* (1) | AFTOL 51 | AY544648 | - |
| *Xylaria hypoxylon* (2) | 303 | NG_027599 | AY544692 |
|  |  |  |  |
|  |  |  |  |
