# Supplementary material for: Extremotolerant fungi from alpine rock lichens and their phylogenetic relationships
Source: Fungal Divers. 2015 Aug 22;76:119–42. doi: 10.1007/s13225-015-0343-8 (PMC4739527; doi:10.1007/s13225-015-0343-8)
Supplement: Supplementary file 8 — Information about sizes of the genetic datasets before and after removing introns and ambiguous SNPs. The mitochondrial locus 16S was not used in the datasets of Leotiomycetes and Sordariomycetes. The original datasets of the nuclear 28S and 18S of Dothideomycetes were retrieved from previously composed datasets (Muggia et al. 2013) already trimmed from introns. (DOCX 13 kb) [file 13225_2015_343_MOESM5_ESM.docx]

**Table S5.** Information about sizes of the genetic datasets before and after removing introns and ambiguous SNPs. The mitochondrial locus 16S was not used in the datasets of Leotiomycetes and Sordariomycetes. The original datasets of the nuclear 28S and 18S of Dothideomycetes were retrieved from previously composed datasets (Muggia et al. 2013) already trimmed from introns.

| **Dataset** | **Genetic loci** | | |
| --- | --- | --- | --- |
|  | **nuLSU 28S** | **nuSSU 18S** | **mtSSU 16S** |
| Chaetothyriales total | 2900 | 4352 | 1374 |
| Chaetothyriales trimmed | 1361 | 708 | 639 |
| Dothideomycetes total | n.a. | n.a. | 855 |
| Dothideomycetes trimmed | 1242 | 705 | 583 |
| Leotiomycetes total | 2306 | 1845 | - |
| Leotiomycetes trimmed | 1355 | 900 | - |
| Sordariomycetes total | 2270 | 1000 | - |
| Sordariomycetes trimmed | 1302 | 927 | - |
